# Sustained Endocytosis Inhibition via Locally‐Injected Drug‐Eluting Hydrogel Improves ADCC‐Mediated Antibody Therapy in Colorectal Cancer

**DOI:** 10.1002/advs.202407239

**Published:** 2024-11-19

**Authors:** Chong Wu, Xiaoting Liu, Rong Liu, Shiyao Song, Zi‐Fan Zheng, Yilin Zeng, Yong Mei, Jing‐Yang Zhang, Qijia Duan, Run Lin, Jin‐Zhi Du, Weiling He

**Affiliations:** ^1^ Department of Gastrointestinal Surgery The First Affiliated Hospital Sun Yat‐sen University Guangzhou Guangdong 510080 China; ^2^ Department of Immunology Zhongshan School of Medicine Sun Yat‐sen University Guangzhou Guangdong 510080 China; ^3^ School of Medicine South China University of Technology Guangzhou Guangdong 510006 China; ^4^ Guangdong Institute for Drug Control NMPA Key Laboratory of Quality Control and Evaluation of Pharmaceutical Excipient Guangzhou 510663 China; ^5^ Department of Pediatrics, The First Affiliated Hospital Sun Yat‐sen University Guangzhou Guangdong 510080 China; ^6^ Department of Radiology, The First Affiliated Hospital Sun Yat‐sen University Guangzhou Guangdong 510080 China; ^7^ School of Biomedical Sciences and Engineering, Guangzhou International Campus South China University of Technology Guangzhou Guangdong 511442 China; ^8^ National Engineering Research Center for Tissue Restoration and Reconstruction South China University of Technology Guangzhou Guangdong 510006 China; ^9^ Department of Gastrointestinal Surgery Xiang'an Hospital of Xiamen University, School of Medicine Xiamen University Xiamen Fujian 361000 China

**Keywords:** antibody‐dependent cell‐mediated cytotoxicity, colorectal cancer, endocytosis inhibition, epidermal growth factor receptor, hydrogel, targeted therapy

## Abstract

The epidermal growth factor receptor (EGFR) is a validated therapeutic target for RAS/RAF wild‐type colorectal cancer (CRC). However, monoclonal antibody‐based anti‐EGFR therapies such as cetuximab have limited effectiveness. Herein, it is identified that EGFR internalization is associated with poor treatment response and prognosis in patients with CRC, based on a retrospective analysis of patients treated with cetuximab. It is further demonstrated that the endocytosis inhibitor prochlorperazine (PCZ) can move EGFR, which is hidden inside the cell, to the cell surface to improve therapeutic antibody binding. Thus, a thermosensitive hydrogel co‐loaded with cetuximab and PCZ (Gel@Cmab/PCZ) is constructed for sustained inhibition of endocytosis and effective cetuximab delivery. Peritumoral injection of Gel@Cmab/PCZ shows strong antitumor efficacy in subcutaneous and orthotopic CRC tumor models and completely abrogated liver metastasis when combined with chemotherapy. In a humanized patient‐derived xenograft model, a single injection of Gel@Cmab/PCZ with one‐third of the conventional cetuximab dose achieved 91% tumor growth inhibition, which promoted NK cell infiltration into tumor tissues and their antibody‐dependent cell‐mediated cytotoxicity effect. This study represents a novel strategy to boost the monoclonal antibody‐mediated anti‐tumor response in CRC.

## Graphical abstract

In this study, a thermosensitive hydrogel loaded with cetuximab and endocytosis inhibitor prochlorperazine is designed to enhance the therapeutic efficacy of colorectal cancer. The hydrogel can sustainably release prochlorperazine at the tumor site, which led to the increased expression of EGFR on the cell membrane, and thus enhances antibody‐dependent cell‐mediated cytotoxicity of cetuximab. This strategy boosts antibody‐mediated anti‐tumor response in multiple tumor models.

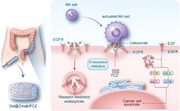

## 1 Introduction

Epidermal growth factor receptor (EGFR)‐blocking monoclonal antibodies (mAbs) (e.g., cetuximab) are the standard therapeutic arsenal for patients with metastatic colorectal cancer (mCRC).^[^ ^1^ ^]^ Anti‐EGFR therapy combined with chemotherapy improves response rates and overall survival in patients with RAS/RAF wild‐type (WT) mCRC.^[^ ^2^ ^]^ However, 30% of patients do not receive the clinical benefits of anti‐EGFR mAb treatment.^[^ ^3^ ^]^ Clinical failure is often attributed to a low initial response rate or rapid drug resistance immediately after treatment.^[^ ^4^ ^]^ In contrast, patients with CRC harboring RAS/RAF mutations (e.g., KRAS G13D) have shown clinical benefits from cetuximab treatment.^[^ ^5^ ^]^ Studies indicate that while RAS/RAF mutation status remains the gold standard for patient selection in clinical practice, it is insufficient to predict the clinical response to cetuximab. Therefore, identifying new biomarkers that can predict the efficacy of anti‐EGFR therapy may significantly enhance clinical outcomes.

Endocytosis of tumor cell‐surface antigens is one mechanism of resistance employed by tumors to drugs that target tumor surface proteins. Surface receptors may be removed from the cell surface before initiating a killing mechanism.^[^ ^6^ ^]^ For instance, the effectiveness of IgG1 therapeutic antibodies, which can initiate antibody‐dependent cellular cytotoxicity (ADCC), relies heavily on their ability to remain enriched on the cell surface for a sufficiently long time. This interaction is crucial for facilitating tumor regression once the Fc region engages with immune effector cells.^[^ ^7^ ^]^ Hence, inhibition of human epidermal growth factor receptor 2 (HER2) internalization increases the effectiveness of the anti‐HER2 antibody trastuzumab against HER2^+^ level of trastuzumab‐mediated ADCC.^[^ ^8^ ^]^ Similarly, rapid internalization of CD20‐rituximab complexes decreases the sensitivity to rituximab treatment, and blocking CD20 internalization overcomes rituximab resistance.^[^ ^9^ ^]^ Recent findings in squamous cell carcinoma (SCC) revealed that EGFR internalization is a significant hurdle to the antitumor activity of cetuximab therapy.^[^ ^10^ ^]^ Endocytosis inhibitors (e.g., prochlorperazine (PCZ)) can facilitate the accumulation and immobilization of EGFR on the tumor cell surface to improve therapeutic antibody binding and the clinical benefit of ADCC‐mediating therapeutic antibodies.^[^ ^10a^ ^]^ The strategy of combining endocytosis inhibitors with therapeutic antibodies is currently undergoing phase I clinical trials for SCC treatment.^[^ ^11^ ^]^ However, the correlation between EGFR internalization and the efficacy of therapeutic antibodies in CRC remains unclear. Moreover, local and continuous delivery of endocytosis inhibitors may further improve EGFR localization at the cell surface and enhance the efficacy of cetuximab treatment.^[^ ^7c^ ^]^

**Scheme 1:**
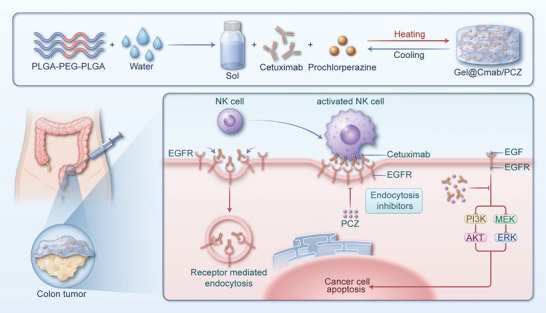
Schematic illustration of the preparation and working mechanism of Gel@Cmab/PCZ. This scheme exhibits the dual mechanism of Gel@Cmab/PCZ therapy in cancer treatment: 1) potentiating cetuximab‐induced ADCC via sustained inhibition of EGFR endocytosis and 2) inhibiting EGFR signaling pathways to induce apoptosis in cancer cells.

Local drug delivery has shown great advantages to increase reginal drug accumulation as well as decrease potential systemic side effects. Clinical trials exploring local administrations for the treatment of CRC have been studied. For examples, oncolytic viruses such as ONCR‐177 (NCT04348916) and VET3‐TGI (NCT06444815), as well as the STING agonist E7766 (NCT04144140) have been employed for the therapy of CRC via localized intratumoral injection. Hydrogel is one of the most important local drug delivery systems for increasing regional drug concentrations and achieving controllable and sustained drug release.^[^ ^12^ ^]^ Therefore, it has been proposed that locoregional delivery of endocytosis inhibitors and therapeutic antibodies may maximize therapeutic benefits. In the present study, we first identified that the location of EGFR expression in patients with CRC could serve as a complementary biomarker for patient selection for cetuximab treatment. EGFR internalization is strongly associated with a poor cetuximab treatment response and prognosis. We further demonstrated that the endocytosis inhibitor PCZ effectively inhibited both EGFR and EGFR‐cetuximab complexes, dramatically increasing the tumor cell‐killing efficacy by enhancing the ADCC effect. Based on this, we constructed an injectable thermoresponsive hydrogel (Gel@Cmab/PCZ) to co‐deliver cetuximab and PCZ for effective CRC treatment. Using cell line‐derived xenograft and patient‐derived xenograft (PDX) models, we systematically verified that this localized delivery strategy could exhibit superior antitumor efficacy over standard clinical treatments, while few abnormalities were observed. Our study provides a simple but potentially translational hydrogel as a drug‐eluting depot to achieve long‐term tumor control in CRC (**Scheme** **1**).

## 2 Results

### 2.1 EGFR Endocytosis is Associated with Poor Response to Cetuximab in Patients with CRC

**Figure 1:**
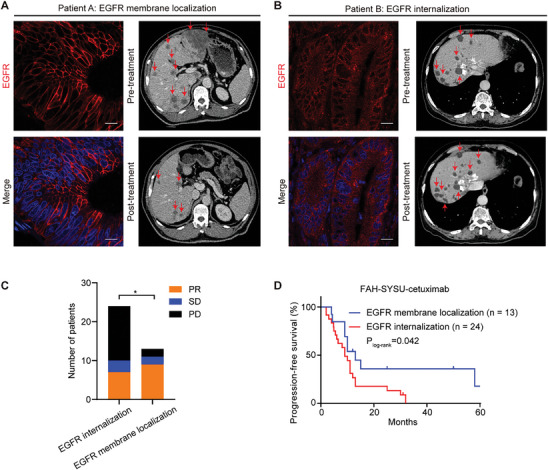
EGFR internalization is associated with poor response to cetuximab in patients with CRC. A, B) Confocal microscopy of human CRC in which (A) EGFR does not undergo internalization (Patient A: EGFR membrane localization) or (B) retains internalization (Patient B: EGFR internalization) and CT scans for Patients A and B were performed pre‐and post‐treatment. Images of the target lesions are shown. Arrows mark the tumor lesions. Scale bars, 15 µm. C, D) Tumor response (C) and progression‐free survival (D) after cetuximab treatment were compared between EGFR membrane localization (*n* = 13) and EGFR internalization (*n* = 24). In C, the statistical difference was derived from the total number of patients in EGFR internalization and membrane localization groups. The Wald chi‐square test (C) and Kaplan–Meier method with log‐rank test (D) were used. *p < 0.05.

In long‐term clonogenic studies, most CRC cell lines are intrinsically resistant to cetuximab, which is consistent with the limited clinical response to cetuximab (Figure S1, Supporting Information). Previous studies have reported that EGFR internalization correlates with patient response to anti‐EGFR treatment.^[^ ^10c^ ^]^ Therefore, we hypothesized that internalized EGFR is associated with cetuximab resistance in patients with CRC. A cohort of 37 patients with CRC who received cetuximab therapy was retrospectively analyzed. Tumor sections from patients with CRC were subjected to EGFR immunofluorescence (IF) staining to explore the relationship between EGFR internalization and cetuximab therapeutic efficacy. Analysis of tumor samples by confocal and fluorescence micrographs clearly showed two primary localization patterns within patient samples: **Figure** **1**A shows a lesion where EGFR does not undergo internalization, and the majority of EGFR proteins are localized to the cell membrane (EGFR membrane localization), whereas Figure 1B shows a typical internalizing EGFR lesion in which the cell endosomes can be observed (EGFR internalization). Samples from 37 patients with CRC who had received cetuximab therapy were analyzed for EGFR localization, and the EGFR trafficking status is summarized in Table S1 (Supporting Information); 24 of 37 (64.8%) patients showed EGFR internalization, whereas 13 of 37 (35.2%) showed EGFR membrane localization.

Patient A, a 67‐year‐old male, was diagnosed with metastatic ascending colon cancer and exhibited EGFR membrane localization. The patients underwent urgent palliative right hemicolectomy due to an incomplete intestinal obstruction. Postoperatively, following one cycle of FOLFIRI chemotherapy, a notable increase in both the size and number of hepatic and pulmonary metastatic lesions was observed. Consequently, the treatment protocol was altered to include cetuximab in combination with chemotherapy. Remarkably, after eight treatment cycles, a significant reduction in the size of the hepatic and pulmonary metastases was observed (Figure 1A; Figure S2, Supporting Information), along with a decrease in tumor markers. This outcome was clinically classified as a partial response (PR). Contrastingly, Patient B (Figure 1B; Figure S2, Supporting Information), showed a typical EGFR internalization lesion, with the fluorescent signal of EGFR distinctly observable in the endosomes. The patient was diagnosed with descending colon cancer with liver metastases and underwent surgical resection. After the procedure, the patient was discharged and continued to receive adjuvant therapy (mFOLFOX6 in combination with cetuximab). Repeat abdomen CT after six treatment cycles revealed greater enlargement of the tumor as well as new metastatic lesions. This outcome was clinically classified as progressive disease (PD). Furthermore, we found that EGFR internalization at tumor sites was associated with high rates of stable disease (SD) and PD (70.8% versus 30.8%, P = 0.036) as well as poor progression‐free survival (PFS) (Figure 1C,D).

### 2.2 PCZ Enhances Surface EGFR Expression Level and mAb‐Mediated Antitumor Activity in Cetuximab‐Resistant CRC Tumor Cells

**Figure 2:**
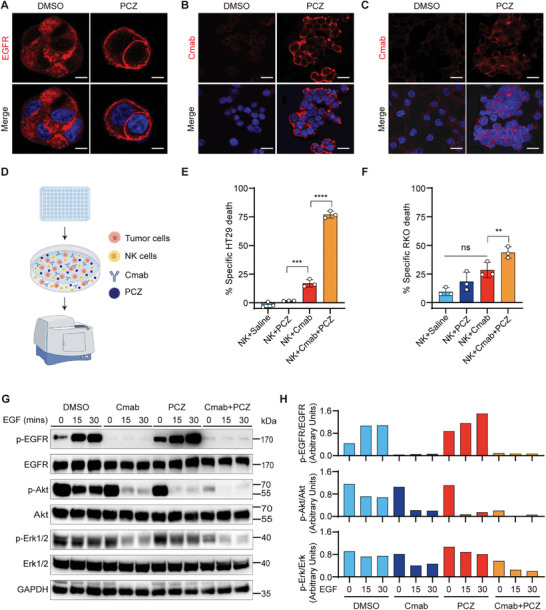
PCZ enhances cetuximab‐mediated ADCC activity and inhibits EGFR downstream pathways in cetuximab‐resistant CRC tumor cells. A) HT29 cells were treated with PCZ at a dose of 10 mM for 24 hours, and EGFR was visualized using confocal fluorescence microscopy. Scale bars, 10 µm. B, C) Immunofluorescence images of HT29 (B) and RKO (C) cells incubated with cetuximab (Cmab) or in combination with PCZ. Scale bars, 20 µm. D) Flowchart of in vitro ADCC experiment. E, F) HT29 (E) and RKO (F) cells were incubated for 6 h with NK cells at a ratio of 50:1 (NK: Tumor cells) in the presence of 100 µg mL^−1^ of Cmab in the absence or presence of PCZ. G) EGFR signaling analysis of HT29 cells treated with Cmab and PCZ. H) Quantitation of immunoblots. Data are presented as mean ± S.D. (*n* = 3). Statistical significance was calculated using one‐way ANOVA (E, F). ns, non‐significant, **p < 0.01, ***p < 0.001, ****p < 0.0001.

To investigate whether EGFR expression in CRCs exhibits the same heterogeneity as in clinical specimens, we assessed the expression and localization of EGFR in various CRC tumor cell lines using IF staining. The results indicated that EGFR expression and endocytosis rates varied in the CRC cell lines **Figure** (**2**A; Figure S3, Supporting Information). Previous studies have confirmed that PCZ inhibits the internalization of PD‐L1 in B16‐F10 cells and EGFR in A431 cells.^[^ ^7^, ^8^, ^10^ ^]^ Therefore, we explored whether PCZ could regulate EGFR localization in CRC cells. HT29 cells were treated with PCZ, and confocal microscopic IF analysis revealed that PCZ localized EGFR to the plasma membrane in HT29 cells compared to that in the untreated control (Figure 2A). High EGFR expression levels are positively correlated with ADCC efficacy.^[^ ^13^ ^]^ Thus, we examined the endogenous expression of EGFR across different human CRC cell lines. Western blot (WB) analysis showed relatively high expression of EGFR proteins in HT29 and RKO cells (Figure S4, Supporting Information). Therefore, these cell lines were selected for subsequent experiments.

ADCC plays a significant role in the antitumor activity of cetuximab, which primarily depends on its capacity to remain bound to the cell surface for a sufficiently long time to allow its Fc region to interact with immune effector cells, thereby facilitating tumor clearance. In HT29 and RKO cells that had not received PCZ treatment, cetuximab was minimally present at the plasma membrane and within internalized structures during the 4‐hour assay period, potentially hindering ADCC activity. In contrast, pre‐treatment with PCZ markedly increased the binding of cetuximab to EGFR on the cell surface (Figure 2B,C). Cetuximab attaches to the ligand‐binding domain of EGFR, and thus EGF‐Alexa^488^ staining can serve as an indirect marker of cetuximab binding locations. Following PCZ treatment of tumor cells, an increase in EGF cell surface staining was observed, indicating a rise in the availability of cetuximab‐binding sites on the cell surface compared to untreated cells (Figure S5, Supporting Information). In the in vitro ADCC assay, only 16.9% and 28.4% of HT29 and RKO cells, respectively, died because of cetuximab‐induced ADCC (Figure 2D–F). Incubation with PCZ increased the cetuximab‐induced ADCC, resulting in 76.9% and 43.7% killing in HT29 and RKO cells, respectively (Figure 2D–F).

An issue arises from blocking endocytic processes, which could enhance the activation of growth‐promoting signaling pathways in cancer cells, potentially leading to tumor growth and treatment resistance.^[^ ^13^, ^14^ ^]^ In HT29 cells, EGFR phosphorylation increased upon EGF stimulation. PCZ treatment increased p‐EGFR, which is consistent with findings by Chew et al.^[^ ^10a^ ^]^ Additionally, the expression of downstream EGFR signaling proteins was detected. p‐Akt and p‐Erk1/2 were observed without ligand stimulation, indicating that the PI3K/Akt and MAPK/ERK pathways were constitutively activated. Cetuximab treatment decreased EGFR phosphorylation; however, the phosphorylation levels of Akt and Erk remained unchanged without EGF stimulation. Consequently, cetuximab alone is ineffective at inhibiting signaling in cetuximab‐resistant cells. Cetuximab combined with PCZ decreased the phosphorylation of both Akt and Erk, reinforcing the signaling inhibitory effect of cetuximab (Figure 2G,H). These results align with previous findings,^[^ ^10^, ^15^ ^]^ which demonstrated that broad‐spectrum endocytosis inhibitors such as PCZ or lovastatin can block downstream signaling pathways during endocytosis inhibition.^[^ ^16^ ^]^ These results suggest that this combined strategy strengthens the antitumor efficacy at multiple levels.

### 2.3 Preparation and Characterization of Gel@Cmab/PCZ Hydrogel

**Figure 3:**
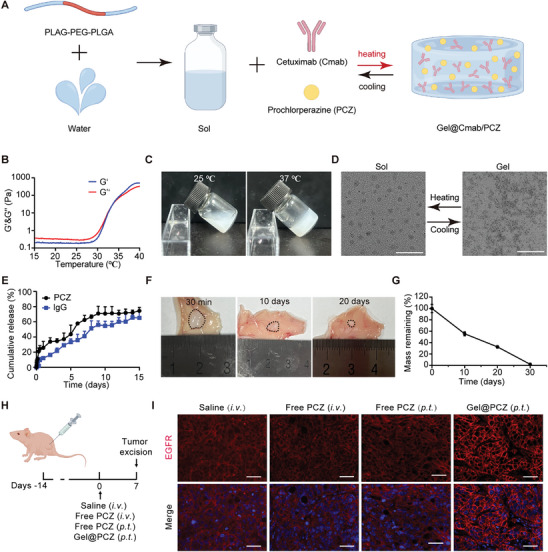
Preparation and characterization of Gel@Cmab/PCZ. A) Schematic illustration of the preparation of Gel@Cmab/PCZ. B) The storage modulus (G’) and loss (G’’) modulus of gel. C) Photographs of the sol‐to‐gel transition with the increasing temperature. D) Representative TEM images of thermo‐sensitive sol‐gel translation (10 wt%). E) Cumulative release profiles of PCZ and Cmab from hydrogels incubated with PBS at pH 7.4 (*n* = 3). F) Representative photographs of gel at different time points after subcutaneous injection in mice. G) Quantitative analysis of remaining weights of gel at 30 min, and on 10, 20, and 30 days (*n* = 3). H) Schematic illustration showing the treatment schedule. *i.v*., intravenous injection; *p.t*., peritumoral injection. I) Immunofluorescence demonstrates the effect of saline, free PCZ (*i.v*.), free PCZ (*p.t*.) and PCZ‐loaded hydrogel (Gel@PCZ) on the inhibition of EGFR endocytosis in vivo (*n* = 3). Scale bar: 50 µm. Data are presented as mean ± S.D. IgG, immunoglobulin G.

Administering drugs locally can decrease drug build up in other organs and enhance the efficiency of delivering therapeutic drugs to the intended target. Thus, we employed a thermosensitive, biodegradable, and biocompatible triblock copolymer poly (lactic‐co‐glycolic acid)‐polyethylene glycol‐poly (lactic‐co‐glycolic acid) (PLGA‐PEG‐PLGA), which can transition from sol to gel at body temperature via ring‐opening polymerization, as a sustained‐release drug scaffold (**Figure** **3**A). The NMR analysis of PLGA‐PEG‐PLGA in CDCl_3_ confirmed the expected structure through well‐attributed resonance peaks of various protons, consistent peak splitting, and integral areas. The average molecular weight of the PLGA‐PEG‐PLGA triblock copolymer, estimated at 5200, was determined by analyzing integral peak areas at 4.80, 3.63, and 1.55 ppm in the ^1^H NMR spectrum (Figure S6A,B, Supporting Information). As shown by the gel permeation chromatography (GPC) results, molecular weight distributions of 1.15 are relatively narrow (Figure S6C, Supporting Information). Subsequently, PLGA‐PEG‐PLGA, PCZ, and cetuximab were dissolved in 1 × PBS to yield a 10 wt% solution, which was then maintained on ice. Rheological testing shows that the storage modulus (G') and loss modulus (G″) remain consistently low and stable from 15 °C to 30 °C, suggesting that the material behaves more fluidly within this temperature range. As the temperature approaches 30 °C, G' and G″ begin to increase sharply, with the rate of increase for G' surpassing that of G″. This behavior is indicative of a gelation or cross‐linking reaction within the hydrogel, resulting in a more solid and elastic structure, characteristic of a “sol‐gel” transition (Figure 3B). Upon heating the PLGA‐PEG‐PLGA gel solution to 37 °C for 2 min, a sol‐to‐gel transition occurred, resulting in a stable, milky white hydrogel named Gel@Cmab/PCZ (Figure 3C).

Building upon the rapid sol‐gel transition observed, we investigated the gelation mechanism. According to the microscopic transmission electron microscope (TEM) imaging (Figure 3D), the amphiphilic PLGA‐PEG‐PLGA copolymer formed micellar particles at 25 °C, and they could aggregate into a network at 37 °C, indicating that the aggregation of PLGA‐PEG‐PLGA micelles contributed to the formation of the hydrogel. To investigate the drug release behavior of Gel@Cmab/PCZ hydrogel in vitro, the samples were immersed in phosphate‐buffered saline (PBS, pH 7.4) at 37 °C. The release profiles of PCZ and Cmab were quantified using high‐performance liquid chromatography (HPLC) and enzyme‐linked immunosorbent assay (ELISA), respectively. The results showed that by day 15 of the release experiment, the cumulative release of PCZ reached 74.4% ± 4.4%, while the cumulative release of Cmab was 65.1% ± 8.8% (Figure 3E), indicating that the hydrogel can serve as a sustained release reservoir for the active ingredients PCZ and Cmab. To assess the gelation and degradation of this thermosensitive hydrogel system in vivo, 100 µL of the 10 wt% polymer solution was subcutaneously injected into the dorsal region of mice. Degradation was observed at 30 min and 10, 20, and 30 days post‐injection. Figure 3F illustrates that a stable, milky white hydrogel formed within 30 min post‐injection and gradually degraded over time, with approximately 30% of the gel remaining after 20 days and dissolving approximately 30 days after injection (Figure 3G). The hydrophobic core of the PLGA‐PEG‐PLGA micelle is composed of PLGA, with a hydrophilic PEG coating, which drives micelle aggregation due to the hydrophobic cores repelling the aqueous solution.^[^ ^17^ ^]^

The in vivo physiological environment is considerably more complicated than the in vitro environment. The mechanical properties of hydrogels are affected by postural alterations, hydrolysis, and enzymatic catalysis. To evaluate the drug retention capacity of hydrogel in vivo, the release properties of the two drugs in the subcutaneous tissue were evaluated. Rhodamine B was used as a substitute for PCZ. After subcutaneous injection of the hydrogel loaded with rhodamine B (Gel@RhB), real‐time fluorescence signals were monitored using IVIS. Compared with free rhodamine B, Gel@RhB demonstrated sustained fluorescence throughout the monitoring period, indicating its superior release properties as a drug reservoir (Figure S7A, Supporting Information). To evaluate the release behavior of cetuximab, fluorescein isothiocyanate (FITC)‐labeled cetuximab was employed to construct a Gel@Cmab‐FITC formulation. The results revealed that unlike the rapid loss observed with free Cmab‐FITC, our hydrogel significantly extended the retention time of Cmab‐FITC in vivo (Figure S7B, Supporting Information).

Considering that this therapeutic approach necessitates the inhibition of endocytosis before or concurrent with antibody binding, both drugs must act simultaneously within tumor lesions. Researchers^[^ ^7c^ ^]^ have suggested that a more targeted and efficient delivery of endocytosis inhibitors to tumors via local injection could enhance the localization of cell surface antigen‐antibody complexes, thereby augmenting their therapeutic efficacy. To assess the prolonged efficacy of the hydrogel in preventing the internalization of tumor antigens in vivo, CRC‐bearing female nude mice were randomly grouped and treated with a single injection of saline (*i.v*.), free PCZ (*i.v*.), free PCZ (*p.t*.), or Gel@PCZ (*p.t*.) when the tumor volume reached 150 mm^3^ (Figure 3H). The mice were euthanized seven days post‐administration, and the tumors were excised for EGFR IF staining. Compared to the group receiving intravenous PCZ, the EGFR in the tumor tissue of the Gel@PCZ group exhibited membrane localization (Figure 3I). This suggests that the PCZ‐loaded hydrogel exerted a sustained inhibitory effect on EGFR endocytosis within the tumor foci.

### 2.4 Anti‐Tumor Activity of Gel@Cmab/PCZ in Subcutaneous Xenograft Model

**Figure 4:**
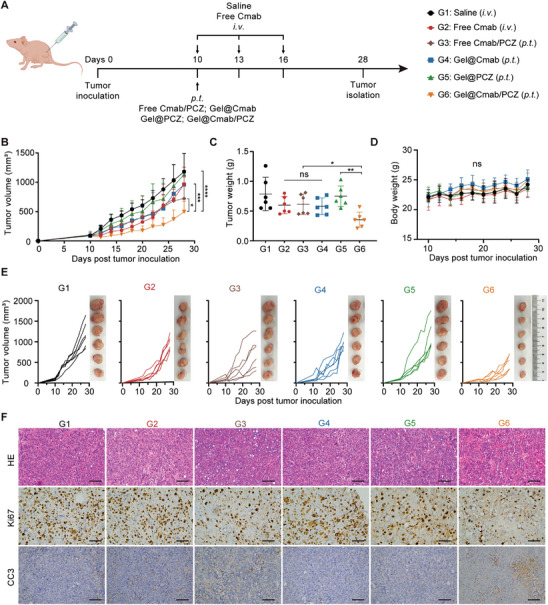
In vivo therapeutic efficacy of Gel@Cmab/PCZ in subcutaneous HT29 tumor‐bearing mice. A) Schematic representation of the antitumor effect in HT29 tumor‐bearing mice. *I.v*., intravenous injection; *P.t*., peritumoral injection. B) Average tumor growth curves of HT29 tumor‐bearing mice after various treatments (*n* = 6). C) The weight of excised HT29 tumor after various treatments (*n* = 6). D) Body weight change curves of mice from different treatment groups at different time points (*n* = 6). E) Image of *ex vivo* HT29 tumors and individual tumor growth with different treatments (*n* = 6). F) Representative H&E, ki67, and cleaved caspase‐3 (CC3) staining of HT29 tumor tissues after different treatments. Scale bars, 50 µm. Data are presented as mean ± S.D. Statistical significance was calculated via two‐way ANOVA (B, D) or the one‐way ANOVA (C). ns, non‐significant, *p < 0.05, **p < 0.01, ***p < 0.001, ****p < 0.0001.

Encouraged by the long‐term inhibitory effect of Gel@PCZ on EGFR endocytosis in vivo, we conducted in vivo experiments to determine whether the combined local delivery of PCZ and cetuximab (Gel@Cmab/PCZ) enhances tumor suppression compared with cetuximab alone (Gel@Cmab) or the free drug. The antitumor efficacy in vivo was assessed by measuring the tumor volume and weight in HT29 xenograft‐bearing nude mice. HT29 cells were implanted subcutaneously in the right flank of the mice. Once the tumors reached 80–110 mm^3^, the mice were divided into six groups to receive various treatments, as shown in **Figure** **4**A. All the mice survived until the end of the experiment. Both the Gel@Cmab (administered via peritumoral injection, a single dose of 1 mg per mouse) and free cetuximab (administered intravenously, once every three days, with each dose being 1 mg per mouse, totaling 3 mg per mouse) groups showed limited tumor‐suppressive effects in the subcutaneous tumor model at approximately 19% relative to saline treatment (Figure 4B–E). In contrast, Gel@Cmab/PCZ exhibited higher tumor treatment efficacy and a lower drug dose than the free cetuximab group. The tumor inhibition rate increased to 58% (P < 0.01 *v.s*. Gel@Cmab, Gel@PCZ, and free cetuximab). This result implies that the local codelivery of PCZ and cetuximab achieved moderate efficacy in HT29 subcutaneous xenograft tumors. The histological analysis of cleaved caspase‐3 (CC3) and Ki67 immunohistochemistry staining indicated that Gel@Cmab/PCZ reduced cancer cell proliferation and increased apoptosis in vivo (Figure 4F). Additional safety assessments of major organs and serum from mice revealed no specific toxicity in either the H&E‐stained organ samples or serum (Figure S8, Supporting Information).

### 2.5 Anti‐Tumor Activity of Gel@Cmab/PCZ Plus Chemotherapy in Orthotopic CRC Tumor Model

**Figure 5:**
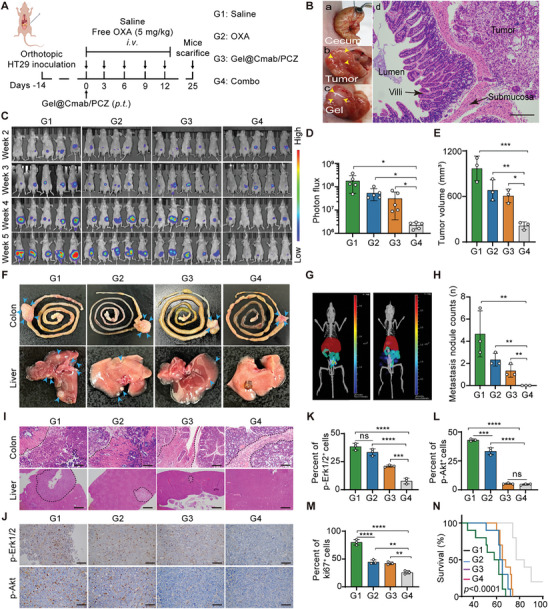
In vivo antitumor efficacy of Gel@Cmab/PCZ plus OXA in orthotopic HT29 tumor model. A) Schematic representation of the antitumor effect in orthotopic HT29 tumor model. The treatments include: G1: Saline, G2: OXA, G3: Gel@Cmab/PCZ and G4: Combo (OXA plus Gel@Cmab/PCZ). *I.v*., intravenous injection; *P.t*., peritumoral injection. B) The technique of cecum wall injection and the confirmation of the orthotopic CRC model. Scale bars, 100 µm. C) In vivo bioluminescence imaging in nude mice with different treatments (*n* = 5). D) Tumor signals were determined by bioluminescence imaging from C (*n* = 5). E) Analysis of tumor volume at the end of treatment (*n* = 3). F) Representative images of primary tumors (blue arrowheads) and liver metastasis (blue arrowheads) in the model. G) Imaging of CRC liver metastasis using integrated in vivo bioluminescence imaging and 3D organ reconstruction. H) Analysis of liver metastasis nodule counts (*n* = 3). I, J) The representative of H&E (I), p‐Erk1/2, and p‐Akt (J) staining images of tumor tissues or liver metastatic foci after different treatments. Scale bars, 50 µm. K–M) Quantitative analysis of immuno‐histochemical staining of p‐Erk1/2^+^ (K), p‐Akt^+^ (L), and ki67^+^ (M) cells in orthotopic tumors of HT29‐Luc orthotopic CRC model under the indicated treatment (*n* = 3). N) Survival of mice from different treatment groups (*n* = 10). Statistical significance was calculated using one‐way ANOVA (D, E, H, K–M) or the log‐rank Mantel‐Cox test (N). ns, non‐significant, *p < 0.05, **p < 0.01, ***p < 0.001, ****p < 0.0001.

Targeted agents combined with chemotherapy are standard treatments for patients with mCRC.^[^ ^2^ ^]^ This approach improves the survival and response rates. However, the treatment shows limited effectiveness. After confirming the effectiveness of Gel@Cmab/PCZ in subcutaneous xenograft models, we further investigated whether the anti‐tumor activity of Gel@Cmab/PCZ is retained in the orthotopic environment and exhibits a combined effect with chemotherapy (**Figure** **5**A). Thus, we developed an orthotopic xenograft model. This orthotopic CRC tumor grew, infiltrated the bowel wall, and spontaneously developed liver metastasis, thereby replicating the metastatic process of human CRC. HT29‐luc cells (2.0 × 10^6^ cells mouse^−1^) were injected into the cecal serosa to establish an orthotopic CRC model (Figure 5B). Fourteen days post inoculation, the mice were assigned into four treatment groups: saline, free OXA, Gel@Cmab/PCZ, or a combined group. Tumor growth was monitored weekly using the Xenogen IVIS Lumina system. Compared to the saline group, OXA or Gel@Cmab/PCZ treatment alone showed limited therapeutic efficacy. Surprisingly, the combination of free OXA and Gel@Cmab/PCZ exhibited dramatic anti‐tumor effects (Figure 5C). At week 5, the mean bioluminescence intensity of the combination treatment group was only 13.1% and 48.3% of those from the mice treated with saline and free OXA, respectively (Figure 5D). As expected, the combined group showed significantly reduced tumor size compared with all the other formulations, with saline as the control (Figure 5E,F).

Liver metastasis remains a major cause of mortality (44). Liver metastasis was measured by the naked eyes, fluorescence imaging, and H&E staining in the orthotopic CRC model. After treatment, the mice were euthanized and underwent examination of their primary tumors and liver. OXA combined with Gel@Cmab/PCZ treatment significantly inhibited orthotopic tumor growth and liver metastasis compared with the other three groups (Figure 5F,G). In the saline group, 80% of mice developed hepatic metastases. In sharp contrast, we found that combination treatment of OXA and Gel@Cmab/PCZ completely eradicated liver metastases, which is markedly better than single treatment with OXA either Gel@Cmab/PCZ (Figure 5H, Table S2, Supporting Information). The effect of treatment was further explored through H&E staining of tumor sections. Distant metastasis begins with tumor invasion. In the control or monotherapy groups, tumors penetrated the mucosal layer and disrupted the integrity of the muscle layer. Histological examination revealed significant metastatic lesions in liver samples. In contrast, in the combination treatment group, the tumors did not penetrate the muscular layer, and no metastatic lesions were detected in the liver (Figure 5I). Cachexia is a common symptom in patients with advanced cancer, who typically display profound weight loss. As the tumor progressed, the body weight of mice in the saline control group decreased, especially in the later stages. In contrast, body weight in the combined treatment group remained stable, indicating better overall health and reduced cachexia in the treated mice (Figure S9, Supporting Information).

To explore the mechanism underlying the antitumor effect of the combination of Gel@Cmab/PCZ and OXA, we performed immunohistochemical (IHC) staining of tumor sections from different treatment groups, focusing on the proliferation marker Ki‐67, apoptosis marker CC3, and markers of the EGFR downstream pathways, p‐Erk1/2 and p‐Akt. Histological analyses demonstrated that the combined group effectively suppressed cell proliferation and the EGFR downstream signaling pathway in tumors (Figure 5J–M; Figure S10, Supporting Information). Furthermore, the combined group exhibited additional survival benefits compared to the single‐agent group. Survival analysis showed that the median survival time increased from 60 days in the control group to 80 days in the combined group (Figure 5N). These outcomes suggest that the co‐administration of cetuximab and PCZ via the hydrogel provides potent synergistic effects against tumor growth and metastasis, reduces cancer‐associated cachexia, and improves survival.

### 2.6 Anti‐Tumor Activity of Gel@Cmab/PCZ in Humanized Patient‐Derived Xenograft (PDX) Model

**Figure 6:**
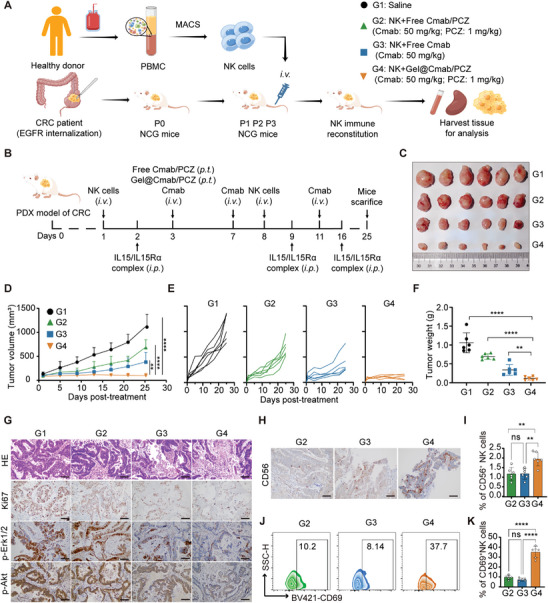
Anti‐tumor activity of Gel@Cmab/PCZ in an EGFR‐internalized CRC PDX tumor model. A, B) Schematic illustration of the experimental design used to evaluate antitumor activity in vivo using partially humanized CRC‐PDX mice (A), which were derived from a sample of patient with primary CRC featuring EGFR internalization and the treatment schedule (B). C) Representative tumor images on day 25 (*n* = 6). D, E) Average and individual tumor growth curves of CRC‐PDX after various treatments (*n* = 6). F) The weight of excised CRC‐PDX after various treatments (*n* = 6). G) Representative H&E, Ki67, p‐Erk1/2, and p‐Akt staining images of tumor tissues after different treatments. Scale bars, 50 µm. H) Representative CD56 staining images of tumor tissues after different treatments. Scale bars, 50 µm. I) The percentage of NK cells (CD56^+^) in isolated tumor tissues after treatment (*n*  =  6). J, K) Representative flow cytometry plots (J) and quantification (K) of the population of CD69^+^ activated NK cells in isolated tumor tissues after treatment (*n*  =  6). Data are presented as mean ± S.D. Statistical significance was calculated using two‐way ANOVA (D) or the one‐way ANOVA (F, I, K). ns, non‐significant, **p < 0.01, ****p < 0.0001.

Receptor internalization reduces antitumor immunity by impairing ADCC activity, which is a key mechanism underlying the clinical efficacy of IgG1 therapeutic antibodies. To assess the capability of Gel@Cmab/PCZ in enhancing ADCC effects in more clinically oriented models, therapeutic studies were carried out on NK cell‐humanized NCG mice with EGFR‐internalization CRC‐PDX model to evaluate ADCC in vivo (**Figure** **6**A). CRC surgical specimens were implanted into NCG mice to establish PDX models. After three generations of passaging, human CRC xenografts from these models were used for ex vivo evaluation. IHC analysis revealed that PDX tissues were typical EGFR‐internalization adenocarcinomas without lymphoma transformation (Figure S11, Supporting Information). NK cells were isolated from a healthy donor, with flow cytometry confirming purity exceeding 90% (Figure S12, Supporting Information). Subsequently, an NK cell immune reconstitution mouse model was established by the intravenous administration of hNKs into immunodeficient NCG mice (Figure S13, Supporting Information).

PDX models were divided into four treatment groups: saline, cetuximab, free Cmab/PCZ, and Gel@Cmab/PCZ (Figure 6B). During the initial stages of conventional treatment (i.e., multiple intravenous administrations of cetuximab), a significant tumor response and reduction in growth rate were observed compared to the control group. This response highlights the early efficacy of the treatment. However, in the intermediate and late phases of the treatment regimen, an accelerated tumor growth rate was noted, suggesting the emergence of drug resistance. In contrast, a single injection of Gel@Cmab/PCZ achieved 91% tumor growth inhibition at doses three times lower than that of cetuximab (*i.v*.) (Figure 6C–F). All mice survived until the end of the study, and tumors harvested on day 25 showed significantly lower proliferation indices (Ki67) and diminished p‐Akt and p‐Erk1/2 expression in the combination therapy group compared to the control group (Figure 6G). IF staining of EGFR revealed a higher localization of EGFR on the cell membrane in the hydrogel treatment group than in the other groups (Figure S14, Supporting Information).

NK cells must infiltrate the tumor tissue to exert a direct cytotoxic effect on tumor cells. We examined NK cell infiltration and activity within tumors to address the effect of Gel@Cmab/PCZ treatment on NK cells. Flow cytometry analysis showed that Gel@Cmab/PCZ treatment significantly enhanced NK cell infiltration at PDX tumor sites. Furthermore, IHC revealed clusters of NK cells close to the tumor foci in the experimental group, in contrast to the dispersed infiltration observed in the control group (Figure 6H,I; Figure S15, Supporting Information). Simultaneously, the expression of the activation marker CD69 in NK cells markedly increased from 9.17% in the control group to 32.92% in the Gel@Cmab/PCZ‐treated group (Figure 6J,K). These data demonstrate that Gel@Cmab/PCZ promoted the infiltration and activation of NK cells within tumor tissues and remarkably suppressed tumor growth in the PDX tumor model.

## 3 Discussion

In clinical practice, patients with RAS/RAF wild‐type CRC can benefit from anti‐EGFR antibody (e.g., cetuximab) therapy. However, 30% of patients do not receive the clinical benefits of anti‐EGFR mAb treatment. Therefore, it is necessary to identify potential biomarkers that can predict the efficacy of cetuximab and develop novel therapeutic strategies to reverse cetuximab resistance in CRC. Joseph et al. validated a reliable method for measuring endocytosis in human tumors and demonstrated that patients with tumor EGFR evading endocytosis responded more favorably to anti‐EGFR mAb treatment.^[^ ^10c^ ^]^ However, this detection method necessitates maintaining the viability of tumor samples for testing, thereby placing greater demands on sample‐handling techniques. Our study revealed that EGFR internalization, which was detected by simple fluorescence staining, was associated with poor treatment response and prognosis in patients with CRC based on a retrospective analysis of patients treated with cetuximab. This indicates that patients with CRC with EGFR localized to the cell membrane exhibit a high response rate to cetuximab. Therefore, the efficacy of cetuximab in the treatment of CRC could be enhanced by regulating EGFR trafficking. A clinical phase I trial was designed to evaluate whether the intravenous infusion of PCZ increased the number of cetuximab‐binding sites on the cell surface.^[^ ^11a^ ^]^ Another phase Ib trial showed that the combination of cetuximab and PCZ at concentrations that could inhibit dynamin exhibited no serious adverse events.^[^ ^11b^ ^]^ These studies demonstrated the potential effectiveness of this combination strategy in the context of CRC. Our data demonstrate for the first time that the endocytosis inhibitor PCZ can inhibit the internalization of EGFR in CRC cells and help it aggregate on cell membranes, which enhances cetuximab‐mediated ADCC activity.

Systemic administration of PCZ has been shown to accumulate in tumor tissues and effectively inhibit endocytosis temporarily.^[^ ^10a^ ^]^ These factors restrict its capacity to augment cetuximab therapy. However, the systemic administration of monoclonal antibody drugs faces challenges owing to their limited permeability and systemic toxicity, thereby reducing drug utilization and limiting dosage optimization. We constructed a thermosensitive hydrogel delivery system based on PLGA‐PEG‐PLGA. During synthesis, PCZ and cetuximab were loaded into the hydrogels (Gel@Cmab/PCZ). In the context of CRC, we demonstrated that hydrogel scaffolds implanted directly at the tumor site could sustainably inhibit EGFR internalization, increase cetuximab binding to tumor cells, and enhance cetuximab‐mediated ADCC activity. The activation of EGFR signaling is critical for cancer progression and chemoresistance in CRC. We found that PCZ collaborates with cetuximab and significantly inhibits EGFR downstream pathways in vitro and in vivo. Gel@Cmab/PCZ plus oxaliplatin effectively suppresses tumor growth and completely abrogates liver metastasis. Consequently, we considered that Gel@Cmab/PCZ could enhance the efficacy of OXA treatment in CRC by inhibiting activation of the EGFR signaling pathway. Overall, this hydrogel‐based local delivery strategy strengthened anti‐tumor efficacy at multiple levels.

Cetuximab resistance has been partly attributed to oncogenic mutations downstream of EGFR in RAS or BRAF. According to the European Society for Medical Oncology (ESMO) and National Comprehensive Cancer Network (NCCN) guidelines,^[^ ^18^ ^]^ patients with these mutations were excluded from cetuximab treatment. In contrast, we demonstrated that a single injection of Gel@Cmab/PCZ induced sustained tumor growth inhibition and achieved 91% tumor growth inhibition at markedly lower doses than cetuximab (*i.v*.) in a CRC PDX model with KRAS mutant. The improved efficacy of Gel@Cmab/PCZ is due to the enhanced delivery of both cetuximab and PCZ to the tumor site, which contributes to enhanced ADCC activity and inhibition of the EGFR downstream pathway. Gel@Cmab/PCZ promoted NK cell infiltration into tumor tissues, which may be attributed to the increased accumulation of anti‐EGFR antibodies on the tumor surface. This accumulation likely resulted from the agglomerative effect of these antibodies, which attracted NK cells to the tumor site. To support this hypothesis, IHC analysis using anti‐CD56 on treated PDX tissues showed a more pronounced clustering of NK cells at tumor sites in the Gel@Cmab/PCZ treatment group, further substantiating our conjecture. Because the humanized CRC‐PDX model better mimics the colorectal tumor growth and treatment response in patients^[^ ^18^, ^19^ ^]^ than orthotopic or subcutaneous cancer models, the efficacy of Gel@Cmab/PCZ obtained using this model may have better clinical translational potential, and thus warrants future testing in clinical trials. Furthermore, the FDA approved the materials and drugs used in the entire delivery system, demonstrating they can be readily available for clinical testing.

## 4 Conclusion

In summary, we demonstrated that EGFR internalization is associated with resistance to cetuximab therapy in CRC and that this relationship can be pharmacologically modulated to improve the effectiveness of EGFR‐targeted therapy in CRCs. We successfully constructed a dual‐drug depot consisting of cetuximab and a PCZ‐loaded thermosensitive hydrogel (Gel@Cmab/PCZ). Owing to the combination of monoclonal antibodies and novel small‐molecule endocytosis inhibitors, Gel@Cmab/PCZ constantly increased the cetuximab membrane presence in tumor cells and effectively induced NK cell infiltration and activation, thereby improving the targeting and ADCC efficacy of monoclonal antibodies. Therefore, these dual‐drug depots offer a promising avenue for CRC treatment, especially for enhancing the targeted therapeutic effects and clinical translation.

## 5 Experimental Section

### Patient Inclusion and Follow‐Up

Patients diagnosed with CRC from The First Affiliated Hospital of Sun Yat‐sen University (FAH‐SYSU), who started to receive Cetuximab therapy from December 2010 to March 2021 were retrospectively enrolled. The inclusion criteria were as follows: (1) a pathological confirmation of CRC; (2) positive expression of EGFR as measured by immunohistochemistry; (3) receiving cetuximab therapy. Exclusion criteria involved: (1) insufficient evaluable target lesions; (2) completion of fewer than two treatment courses; (3) absence of tumor response evaluation. A total of 37 patients met the inclusion criteria. The study collected data including follow‐ups, CT scans, pathology reports, and cetuximab response assessments from the hospital's tracking system. The primary endpoint for PFS was determined by either tumor progression or the last follow‐up. Clinical responses to tumors were classified as PR, SD, or PD by radiologists. All study procedures involving human participants and materials complied with the ethical standards of the FAH‐SYSU Ethics Committee, adhering to the 1964 Helsinki declaration and its subsequent updates, or similar ethical standards. Informed consent was obtained from all participants in the study. This study's reporting conforms to the STROBE guidelines.

### Materials

Invitrogen (USA) provided Epidermal growth factor labeled with biotin, bound to Alexa Fluor™ 488 streptavidin (known as Alexa Fluor™ 488 EGF complex, #E13345), Goat anti‐Human IgG (H+L) Cross‐Adsorbed Secondary Antibody (Alexa Fluor™ 555, #A21433), Goat anti‐Rabbit IgG (H+L) Highly Cross‐Adsorbed Secondary Antibody (Alexa Fluor™ 555, #A21429), ProLong™ Gold antifade reagent (#P10144), and Penicillin‐Streptomycin (#15 070 063).Antibodies against EGFR (#4267), Phospho‐EGFR (Tyr1068) (#3777), Ki‐67 (#9449), cleaved caspase‐3 (#9661s), CD56 (#99 746), Akt (#4691), p44/42 MAPK (Erk1/2) (#4695), Phospho‐Akt (Ser473) (#4060), Phospho‐p44/42 MAPK (Erk1/2) (Thr202/.Tyr204 (#4370), pancytokeratin (#4545S), and GAPDH (#2118) were acquired from Cell Signaling Technology (CST) in the United States. Antibodies anti‐human CD45 (#PR70101), and human CD20 (#M0755) were purchased from DAKO (Denmark). Cetuximab (Erbitux) was obtained from the FAH‐SYSU Pharmacy. Gibco provided Roswell Park Memorial Institute‐1640 (RPMI‐1640) medium (#11 875 093), Dulbecco's modified eagle medium (DMEM, #11 965 092), and fetal bovine serum (FBS). BioLegend (San Diego, CA, USA) provided Brilliant Violet 421™ anti‐human CD69 Antibody (#310 929), Brilliant Violet 510™ anti‐human CD45 Antibody (#368 525), Brilliant Violet 650™ anti‐human CD3 (#300 467), and Cell Activation Cocktail (with Brefeldin A). Purchased from PeproTech, Inc. in Rocky Hill, NJ, USA were Recombinant Human IL‐15 (rhIL‐15, #Z03300) and Recombinant Human IL‐2 (rhIL‐2, #200‐02). The Recombinant Human IL‐15R alpha Fc Chimera Protein (IL‐15R alpha, #147‐IR‐100) was purchased from R&D Systems in Minneapolis, MN. Various reagents and solutions including Red Blood Cell Lysis Buffer, DAPI, BCA protein assay kit, immunostaining permeabilization solution, RIPA lysis buffer, Protease and phosphatase inhibitor cocktail, SDS‐PAGE protein sample loading buffers, protein ladder, skim milk powder, BSA, and primary antibody dilution buffer were acquired from Beyotime in Shanghai, China. The Cell Counting Kits‐8 (CCK‐8, #HY‐K0301) were acquired from MedChem Express (MCE) in China. 1×Tris‐Glycine transfer buffer (#G2145‐1L), 1×Tris‐glycine SDS‐PAGE electrophoresis buffer (#G2149‐1L), and Powered tris‐buffered saline with tween 20 (TBST, #G2150‐1L) was purchased from were purchased from Seivicebio (Wuhan, China). The collagenase V (#C9263), DNase I (#DN25), Dispase (#D4693) and Prochlorperazine dimaleate salt (PCZ, #P9178) were purchased from Sigma‐Aldrich (Sigma Aldrich, USA). The collagenase D (product number 11 088 866 001) and lactate dehydrogenase (LDH) cytotoxicity assay kit (Cytotoxicity Detection Kit Plus, product number 4 744 934 001) were acquired from Roche, a company based in Germany. The water utilized in this research was ultrapure and had an electrical resistivity of up to 18.2 MΩ cm^−1^, obtained through the utilization of a laboratory water purification system (Milli‐Q Integral 3, Merck Millipore, Germany).

### Fabrication and Characterization of Drug‐Loaded Hydrogel

*Synthesis and Characterization of PLGA‐PEG‐PLGA*: Polyethylene glycol (PEG) and anhydrous toluene were combined in a 100 mL round‐bottom flask. Water was removed through atmospheric distillation, followed by further dehydration using a vacuum pump. Subsequently, D, L‐lactic acid (D, L‐LA), along with glycolic acid (GA), were introduced into the desiccated flask and dehydrated while under reduced pressure. The dehydrated PEG, D, L‐LA, and GA were then transferred to a glove box and stirred at 130 °C for 0.5 hours. Afterward, a catalyst of stannous octoate (0.2 wt%) was introduced, followed by stirring the mixture at 130 °C for a duration of 12 hours. Post‐reaction, the product was removed from the glove box and unreacted monomers were eliminated under vacuum at the same temperature. The crude polymer that was produced was dissolved in chilled water (4 °C–8 °C) and then heated to 80 °C in order to cause the polymer t4 o precipitate. This precipitated polymer was isolated from the supernatant through chromatography and lyophilized to yield a pale yellow colloidal PLGA‐PEG‐PLGA. For analytical purposes, the PLGA‐PEG‐PLGA was dissolved in CDCl_3_ for NMR hydrogen spectrum analysis and in tetrahydrofuran for GPC analysis.

*Preparation of Gel@Cetuximab/PCZ Drug‐Loaded Gel*: The dosages of cetuximab^[^ ^14h^ ^]^ (1 mg) and prochlorperazine^[^ ^10a^ ^]^ (PCZ, 20 µg) were selected based on previously studies. Cetuximab and PCZ were added to a phosphate‐buffered saline (PBS) solution, followed by their introduction into a 10% concentration sol state of PLGA‐PEG‐PLGA. This mixture was then stirred on ice for 24 hours to achieve a uniformly dispersed drug‐loaded sol. The resultant fluid‐state drug‐loaded sol was drawn into a syringe and stored on ice for subsequent use.

*PCZ and Cmab Release from the Hydrogel*: Release studies were performed at 37 °C with constant agitation in PBS. The released PCZ was analyzed by HPLC, and the antibody release was determined by the Human IgG Precoated ELISA Kit (Dakewe, China).

### In Vivo Degradation of Hydrogel

Initially, a 10% concentration gel solution was stored at 4 °C to maintain it in a fluid sol state. This solution was then drawn into a syringe and kept on ice for subsequent use. Subcutaneously, 100 µL of gel was implanted in the back of each mouse, with three gel pieces implanted per mouse, involving a total of six mice. Following implantation, the gel was removed from the subcutaneous area of the mice's backs at predetermined intervals (30 minutes, 10, 20, 30 days). The weight of the gel was measured, and photographs were taken to document the state of the gel.

### Cell Culture

Prof. RanYi Liu from Sun Yat‐sen University Cancer Center generously provided the human colon cancer cell lines (HCT116, DLD1, HCT15, SW1116, HT29, SW480, RKO, LS174T, SW837), which were then cultured following CTCC guidelines. Cells were cultured in a Heracell™ VIOS 160i CR CO_2_ incubator from Thermo in the USA, with a humidified atmosphere of 5% CO_2_ at 37 °C.

### Preparation of Human NK Cells

Human PBMCs from one healthy donor (40 years old, male adult Asiatic) were obtained from oricells (Shanghai Oribiotech Co., Ltd). Following the receipt of PBMCs, NK cells were purified using the Human NK Cell Enrichment Kit (catalog number 17 955, STEMCELL Technologies) via negative selection. To begin, 50 µL mL^−1^ of the enrichment mixture was added to the cell suspension, which had a density of 5.50 × 10^7^ cells mL^−1^, and the mixture was allowed to incubate at room temperature for 10 minutes. Following this, 100 µL mL^−1^ of magnetic particles were introduced, and incubation continued for an additional 10 minutes at room temperature. The suspension was subsequently placed into the EasySep magnet for another 10 minutes. After this period, the purified NK cells were collected and transferred to a fresh tube, separating them from the remaining cell population (Figure S12, Supporting Information).

### Mice

The Biomedical Model Experimental Animal Center of South China University of Technology bred BALB/c nude and NOD/ShiLtJGpt‐Prkdc^em26Cd52^Il2rg^em26Cd22^/Gpt (NCG) mice. BALB/c nude and NCG mice were purchased from Jiangsu GemPharmatech in Nanjing, China. The study was conducted following an approved protocol from the appropriate committee (No.2022041), adhering to all applicable ethical regulations.

### Long‐Term Clonogenic Assays

Cells were propagated and plated in 6‐well plates at densities ranging from 10,00 to 30,00 cells per well, tailored according to their proliferation rates. These cells were then maintained in a medium infused with specified drugs for a duration of 10 to 14 days. Following the incubation period, cells were fixed using a 4% formaldehyde solution in PBS and subsequently stained with a 0.1% solution of crystal violet in water.

### Immunofluorescence

CRC cells were cultured on coverslips in 24‐well plates and allowed to incubate overnight until they reached 80% confluence. Concurrently, the cells were treated with 60 mg mL^−1^ of cetuximab and 15 mM of prochlorperazine for a period of 4 hours. Following treatment, cells were washed thrice with ice‐cold PBS and then fixed using 4% paraformaldehyde (PFA) in PBS for 20 minutes. This step was succeeded by a 10‐minute permeabilization using 0.1% Triton X‐100 in PBS (PBTX), and cells were subsequently blocked with 2% (w/v) bovine serum albumin (BSA) in PBS. Humanized monoclonal antibodies on the cells were detected by treating them with a secondary antibody (goat anti‐human IgG Alexa Fluor^647^, Life Technologies) for one hour, another round of blocking with 2% BSA in PBS (Sigma‐Aldrich), and staining with DAPI for 5 minutes. Finally, the coverslips were mounted on slides using ProLong™ Gold antifade reagent (Life Technologies) and images were captured with a Zeiss LSM 880 Airyscan confocal microscope (Olympus Life Science) employing a 63 × objective.

### In Vitro Validation of ADCC Effect

Cells were seeded at a ratio of 50:1, with effector cells (NK cells) to target cells (CRC). The seeding was done in serum‐free media supplemented with 0.1% bovine serum albumin (BSA). Treatment involved administering 100 µg mL^−1^ Cetuximab, under conditions both with and without the presence of PCZ. Following a 6‐hour incubation period, cell death was assessed by measuring lactate dehydrogenase (LDH) release using the Cytotoxicity Detection Kit Plus (LDH, Roche).

### In Vitro Dynamin Inhibitor Treatment and EGF Stimulation

To signal analysis, cells were seeded in 10‐cm tissue culture‐treated dishes at an approximate density of 1 × 10^6^ cells per dish. The cells underwent serum starvation for three hours prior to exposure to the designated drugs: cetuximab at a concentration of 60 mg/mL and PCZ at 15 mM, with DMSO serving as the vehicle control. The cells received a 30‐minute drug treatment before and during EGF activation. EGFR activation was conducted using 10 ng mL^−1^ of Alexa Fluor488‐conjugated EGF (Life Technologies) at intervals of 0, 15, and 30 minutes. In combination therapy scenarios, cetuximab was administered five minutes before the introduction of endocytosis inhibitors. After treatment, cells were rapidly washed with ice‐cold PBS and immediately frozen using liquid nitrogen. Lysis was performed using radioimmunoprecipitation assay (RIPA) buffer enhanced with a protease and phosphatase inhibitor cocktail, following the manufacturer's instructions.

For fluorescence analysis post‐EGF stimulation, HT29 and RKO cells were cultivated on coverslips in 24‐well plates and cultured overnight until reaching 80% confluence. Following a three‐hour period of serum starvation, the cells were treated with 15 mM PCZ and DMSO as a vehicle control. For EGF stimulation, the cells were treated with 10 ng mL^−1^ of Alexa Fluor488‐conjugated EGF (Life Technologies) and maintained under continuous drug exposure for 15 minutes at 37 °C. Immediate washing with ice‐cold PBS was done post‐treatment, and the cells were fixed using 4% PFA/PBS for 15 minutes. An additional trio of washes preceded counterstaining with DAPI (50 mM, Life Technologies, Invitrogen) for 10 minutes. Imaging was performed with a Zeiss LSM 880 Airyscan confocal microscope (Olympus Life Science) using a 63 × objective.

### Immunohistochemical Staining

Immunohistochemistry (IHC) assays were conducted utilizing established protocols. At first, FFPE samples from patients with CRC were tested using an EGFR antibody (4267, Cell Signaling Technology). Similarly, FFPE samples from HT29 and CRC‐PDX xenografted tumors were analyzed using a variety of antibodies, such as those that target Ki‐67 (9449, Cell Signaling Technology), cleaved caspase‐3 (9661s, Cell Signaling Technology), CD56 (99 746, Cell Signaling Technology), Phospho‐Akt (Ser473) (4060, Cell Signaling Technology), EGFR (4267, Cell Signaling Technology), and Phospho‐p44/42 MAPK (Erk1/2) (Thr202/Tyr204) (4370, Cell Signaling Technology). Following the primary antibody application, detection of antigen‐positive cells was achieved using DAB+ chromogen. The quantification of staining was accomplished with ImageJ software, evaluating both the percentage of cells showing positive staining and the intensity of staining across high‐power fields in selected sections. Additionally, the H‐score method was applied for EGFR membrane staining, categorizing staining into four levels: 0 indicating no staining, 1+ denoting faint staining visible under high magnification, 2+ reflecting moderate staining, and 3+ for intense linear membrane staining apparent even under low magnification.

### Animal Studies

To create the subcutaneous human CRC model, 100 µL of HT29 cell suspension (2.0 × 10^7^ cells in 1 mL PBS with 20% Matrigel) was injected under the skin on the right side of nude mice. When the tumors reached a size of 80–100 mm^3^, the mice were split into six categories: saline, cetuximab without charge, Cmab/PCZ without charge, Gel with Cmab, Gel with PCZ, and Gel with Cmab/PCZ. Intravenous injections of saline and free‐cetuximab (1 mg per mouse) were administered into the tail vein on days 10, 14, and 18. Each mouse received a single injection around the tumor of free Cmab/PCZ, Gel@Cmab, Gel@PCZ, or Gel@Cmab/PCZ, with cetuximab administered at a dose of 1.0 mg per mouse and PCZ at 0.8 mg k^−1^g. The dimensions of each subcutaneous tumor, including length (L) and width (W), were regularly measured to determine the tumor volume (V) using the formula V = L × W^2^/2. Tumors were collected on the 28th day for analysis of tumor weight, IHC staining, and H&E staining.

To establish an orthotopic colorectal liver metastasis mouse model,^[^ ^20^ ^]^ 2.0 × 10^6^ HT29‐luc cells in a mix of 50.0 µL medium and Matrigel (4:1 ratio) were injected in the cecal wall of nude mice using laparotomy under anaesthetics. Following a two‐week period, the mice were rearranged according to the luminescent signals and administered with saline, Oxaliplatin, Gel@Cmab/PCZ, or a mix of Oxaliplatin and Gel@Cmab/PCZ as specified in the figure caption. Subsequently, the advancement of the tumor was observed through an IVIS Lumina imaging system from PerkinElmer, utilizing Living Image 4.7.4 software, and the measurement of fluorescence signals was assessed using Living Image 4.7.4. Animal weights were recorded regularly.

For the CRC‐PDX model, female NCG mice (6‐8 weeks old, Strain NO. Mice (T001475) were acquired from GemPharmatech in Nanjing, China and raised in conditions free of specific pathogens. A PDX model was created to assess the in vivo effectiveness of Gel@Cmab/PCZ in therapeutic ADCC. A clinical sample (rectal adenocarcinoma, EGFR internalized conditions, Microsatellite Stable) was collected during surgical resection at The First Affiliated Hospital of Sun Yat‐sen University. Following the acquisition of the new sample, the tumor tissue was sliced into 3 × 3 × 3 mm pieces and then inserted under the skin of the NCG mice, with 2–3 pieces per mouse. Following three passages of engraftment, the tumor tissue exhibited positive staining for human EGFR expression and was then transplanted into NCG mice (6 per group) in 2 mm by 2 mm sections. Fourteen days post‐transplantation, newly extracted NK cells (1.0 × 10^6^ cells in 100 µL PBS) were given via injection into the tail vein. In vivo, the IL‐15/IL‐15Rα complex was employed for the expansion and activation of NK cells. Following the NK cells tail vein injection, the IL‐15/IL‐15Rα complex was given intraperitoneally once a week at a dosage of 1.25 µg per mouse. Mice were randomly grouped into treatment cohorts (n = 6 per group): saline, NK+Free Cetuximab (*i.v*.), NK+Free Cmab/PCZ (*p.t*.) and NK+Gel@Cmab/PCZ (*p.t*.). Regular measurements were taken of the size of the tumor and the weight of the animals.

### Biological Safety Evaluation

To assess the safety of this therapeutic interventions, particularly those involving implant materials, an automated biochemistry analyzer (Model 3100, Hitachi, Japan) was utilized for serological testing. Serum for these tests was obtained by centrifuging at 1000 g for 20 minutes at 4 °C. Subsequently, histological analyses of the collected organs were performed, including H&E staining and microscopic examination.

### PDX Genetic and Immunohistochemical Validation

Given the frequent occurrence of patient‐derived EBV‐positive lymphomas in PDX models involving NCG mice, it was crucial to validate that the PDXs accurately reflect the original tissue. To ensure this, H&E and IHC‐stained slides were examined by a pathologist to rule out lymphoma in the PDX models. Formalin‐fixed paraffin‐embedded specimens from CRC‐PDX xenografted tumors were analyzed using an automated Leica Bond‐MAX stainer, employing antibodies against human pancytokeratin (4545S, Cell Signaling Technology), human CD45 (Dako, PR70101), and human CD20 (M0755, Dako). The presence of carcinomas was established through IHC, indicated by pancytokeratin positivity and the absence of CD45 and CD20 staining.

### Histologic Evaluation

Post‐treatment tumors were fixed in 10% neutral formaldehyde for 48 hours at room temperature. This was followed by dehydration through a series of graded ethanol solutions and subsequent embedding in paraffin. The embedded tissues were sectioned into 6 µm slices using a paraffin microtome (RM 2235, Leica, Germany). These sections were then subjected to a melting process at 60 °C, dewaxed in xylene, and rehydrated using reverse‐graded ethanol solutions before being stained with H&E.

### Flow Cytometry Analysis

Tumor samples were finely chopped and then subjected to enzymatic digestion using a solution composed of 10 mM HEPES, 1.25 mg ml^−1^ collagenase D, 0.85 mg ml^−1^ collagenase V, 50 µg ml^−1^ DNase I, 1 mg ml^−1^ Dispase II, 100 U ml^−1^ penicillin, 100 µg ml^−1^ streptomycin, and 10% FBS. The cells liberated from this process were subsequently utilized for FACS analysis. Regarding the in vitro experiments, cells that had undergone co‐culture or stimulation were harvested for the purpose of assessing NK‐cell activation. The analysis was conducted using the BD FACSCelesta (BD Biosciences) according to its standard operating procedures, and data were analyzed with FlowJo software.

To evaluate NK cell activation within the tumors, cells isolated from the tumors were first exposed to a Cell Activation Cocktail (including Brefeldin A) for four hours. Following activation, these cells were labeled with anti‐human CD56, anti‐human CD3, and anti‐human CD69 antibodies from BioLegend. For detection, PE‐conjugated, Brilliant Violet 785‐conjugated, and Brilliant Violet 421‐conjugated secondary antibodies were employed.

### Western Blotting

Cell disruption was achieved by utilizing RIPA buffer, followed by centrifugation of the resulting lysates at 12000 rpm for 30 minutes at 4 °C. After that, the supernatants were gathered and their protein levels were measured with a BCA protein assay kit. The lysates underwent SDS‐PAGE and were subsequently transferred onto PVDF membranes (Merk Millipore, IPVH00010). Primary antibodies such as anti‐EGFR, p‐EGFR, Akt, p‐Akt, Erk1/2, p‐Erk1/2, and GAPDH (all from Cell Signaling Technology) were used in the immunoblotting procedure. Horseradish peroxidase‐conjugated secondary antibodies, either anti‐rabbit/mouse IgG, aided in the detection process.

### Ethical Compliance

Individuals diagnosed with CRC at The First Affiliated Hospital of Sun Yat‐sen University (FAH‐SYSU) in Guangzhou, China, and who initiated cetuximab therapy between December 2010 and March 2021 were selected for retrospective analysis. The inclusion criteria included adherence to all pertinent ethical guidelines and approval by the Ethics Committee of FAH‐SYSU. Each participant had granted written informed consent both for their involvement in the research and for the dissemination of the findings.

### Statistics

The analysis of data was carried out with the help of SPSS 19.0 in Chicago, IL, and GraphPad Prism 6 in San Diego, CA. Continuous variables were presented as means, and discrete variables as medians. Percentages were used to describe categorized variables. The Shapiro‐Wilk test assessed normality of distributions. Data that conforms to a Gaussian distribution were compared between two groups using Student's t‐test, with mean values and standard deviations (mean ± SD) shown as error bars. For comparisons among more than two groups, one‐way or two‐way ANOVA was applied, followed by post‐hoc tests where appropriate. When data deviated from a Gaussian distribution, the Mann‐Whitney U rank‐sum test was used to display median values and ranges with error bars. Categorical differences were analyzed using the Wald chi‐square test. PFS distributions were evaluated with the Log‐rank test, employing Kaplan‐Meier methods. The hypothesis testing was conducted with a two‐sided approach, where a P value below 0.05 was deemed to show statistical significance.

## Conflict of Interest

The authors declare no conflict of interest.

## Author Contributions

C.W. and X.L. contributed equally to this work. W.H., J.D., R.L., conceived and supervised the project. C.W., X.L., R.L., S.S., and Y.Z. collected, analyzed, and interpreted data. R.L., S.Y., Z.Z., Y.M., J.Z., Q.D., and C.W. performed bioinformatic and computational analyses. W.H., J.D., R.L., X.L., and C.W. prepared the manuscript. All authors discussed the results and commented on the manuscript.

## Supporting information



Supporting Information

## Data Availability

The data that support the findings of this study are available from the corresponding author upon reasonable request.
