# Supplementary material for: Sustained Endocytosis Inhibition via Locally‐Injected Drug‐Eluting Hydrogel Improves ADCC‐Mediated Antibody Therapy in Colorectal Cancer
Source: Adv Sci (Weinh). 2024 Nov 19;12(2):2407239. doi: 10.1002/advs.202407239 (PMC11727399; doi:10.1002/advs.202407239)
Supplement: Supplementary file 1 — Supporting Information [file ADVS-12-2407239-s001.docx]

Supporting Information for

Sustained Endocytosis Inhibition *via* Locally-Injected Drug-Eluting Hydrogel Improves ADCC-Mediated Antibody Therapy in Colorectal Cancer

*Chong Wu^#^, Xiaoting Liu^#^, Rong Liu, Shiyao Song, Zi-Fan Zheng, Yilin Zeng, Yong Mei,* *Jing-Yang Zhang,* *Qijia Duan, Run Lin*, Jin-Zhi Du*, Weiling He**

C. Wu, X. Liu, Z. Zheng, Y. Mei, W. He

Department of Gastrointestinal Surgery, The First Affiliated Hospital, Sun Yat-sen University, Guangzhou, Guangdong 510080, China.

Email: [wlhe@xah.xmu.edu.cn](mailto:wlhe@xah.xmu.edu.cn)

R. Liu, Q. Duan, J. Du

School of Medicine, South China University of Technology, Guangzhou, Guangdong 510060, China.

Email: [djzhi@scut.edu.cn](mailto:djzhi@scut.edu.cn)

R. Liu

Guangdong Institute for Drug Control, NMPA Key Laboratory of Quality Control and Evaluation of Pharmaceutical Excipient, Guangzhou 510663, China

W. He

Department of Gastrointestinal Surgery, Xiang'an Hospital of Xiamen University, School of Medicine, Xiamen University, Xiamen, Fujian 361000, China.

J. Zhang

School of Biomedical Sciences and Engineering, Guangzhou International Campus, South China University of Technology, Guangzhou, Guangdong 511442, China.

X. Liu

Department of Immunology, Zhongshan School of Medicine, Sun Yat-sen University, Guangzhou, Guangdong, 510080, China.

J. Zhang, J. Du

National Engineering Research Center for Tissue Restoration and Reconstruction, South China University of Technology, Guangzhou, Guangdong 5100006, China.

S. Song

Department of Pediatrics, The First Affiliated Hospital, Sun Yat-sen University, Guangzhou, Guangdong, 510080, China.

Y. Zeng, R. Lin

Department of Radiology, The First Affiliated Hospital, Sun Yat-sen University, Guangzhou, Guangdong, 510080, China.

Email: [linrun5@mail.sysu.edu.cn](mailto:linrun5@mail.sysu.edu.cn)

**Supporting Figures**


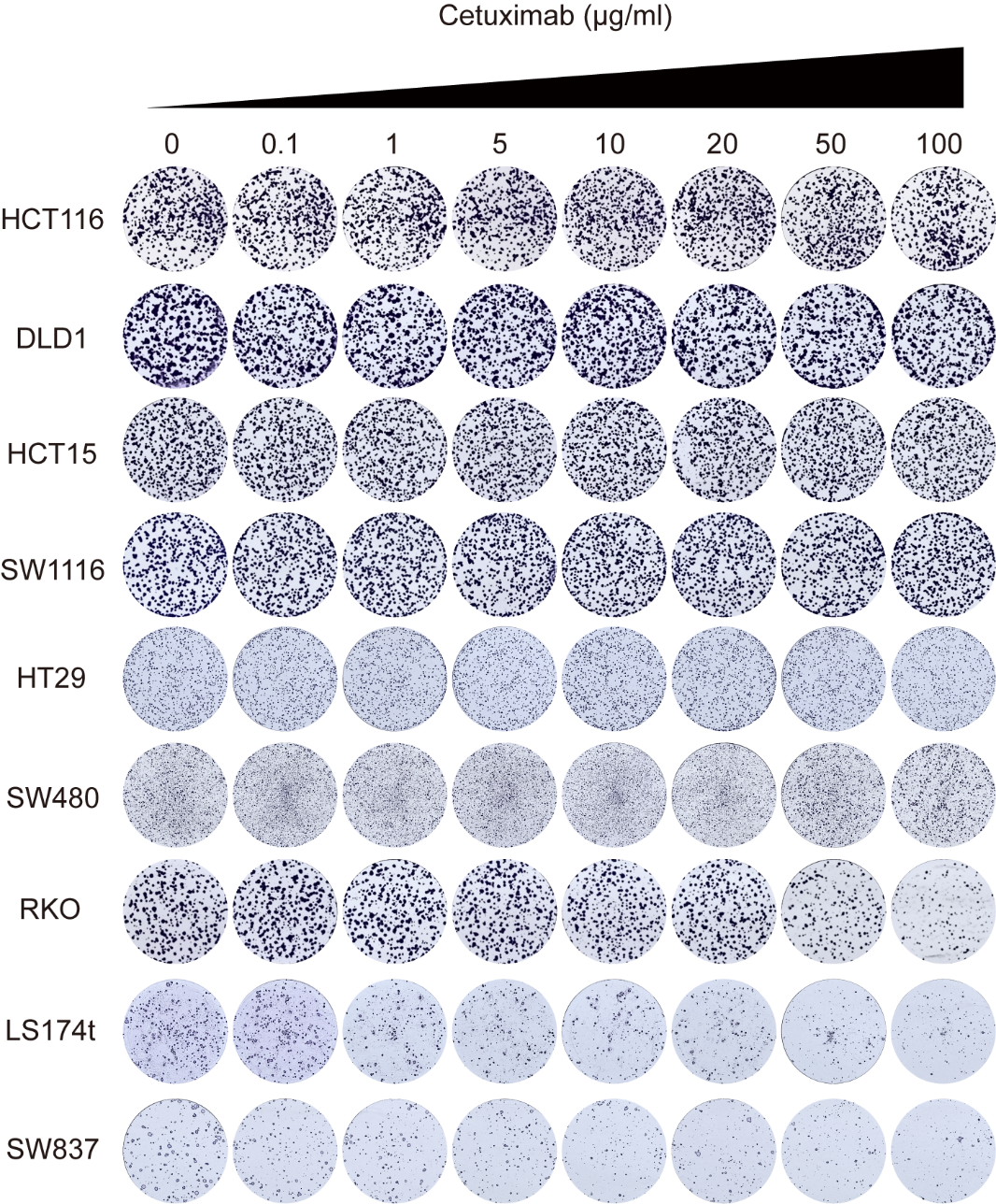


**Figure S1.** CRC cells are resistant to cetuximab treatment *in vitro*. Cells were grown in the absence or presence of cetuximab at the indicated concentrations for 10-14 days, fixed, and stained.


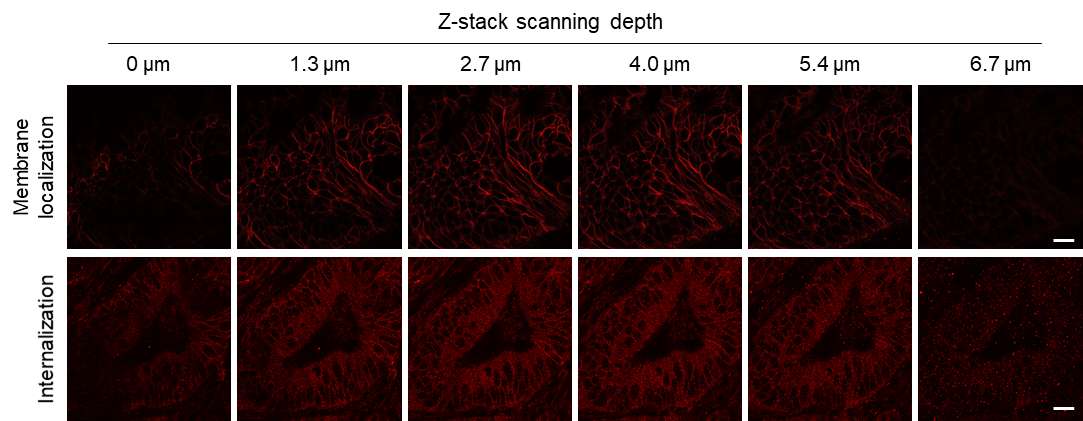


**Figure S2.** Z-stack view of human CRC in Figure 1A and B. Scale bars, 10 μm.


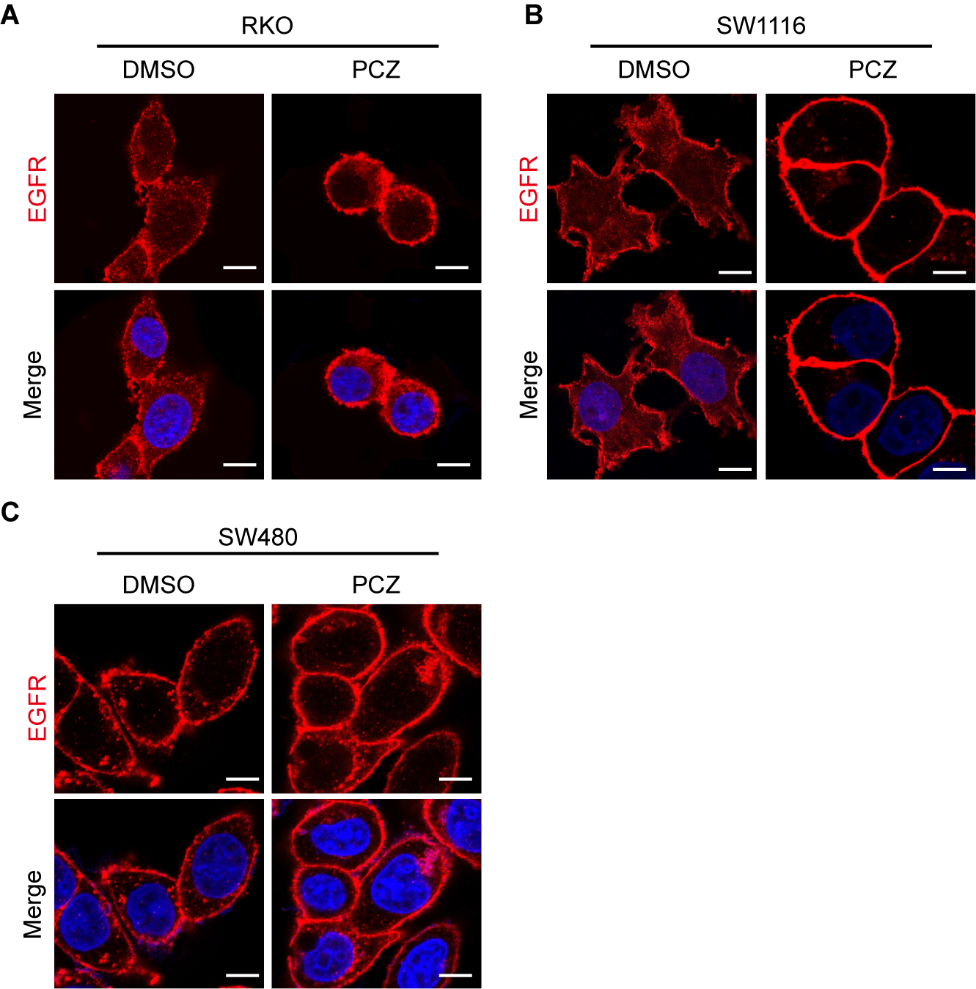


**Figure S3. A-C)** RKO (**A**), SW1116 (**B**) and SW480 (**C**) cells were treated with PCZ at a dose of 10 mM for 24 hours, then EGFR was visualized by confocal fluorescence microscopy.


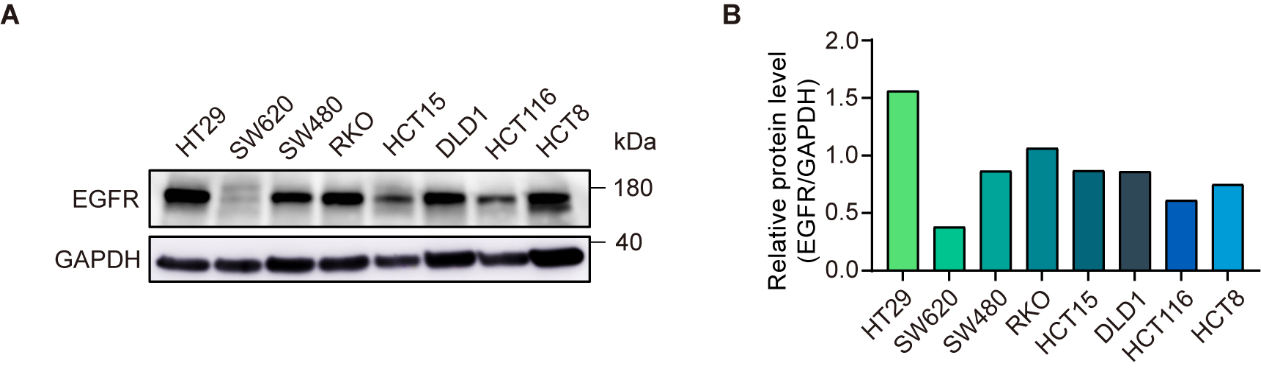


**Figure S4.** A) Western blot analysis of total EGFR levels in a panel of colorectal cancer cell lines. B) Quantitation of immunoblots in (A).


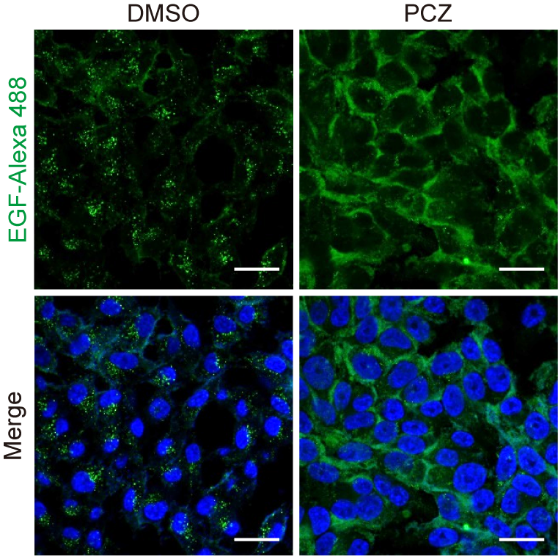


**Figure S5.** Inhibitory effects of PCZ on EGFR ligand-induced endocytosis in CRC cells. Scale bars, 20 μm.


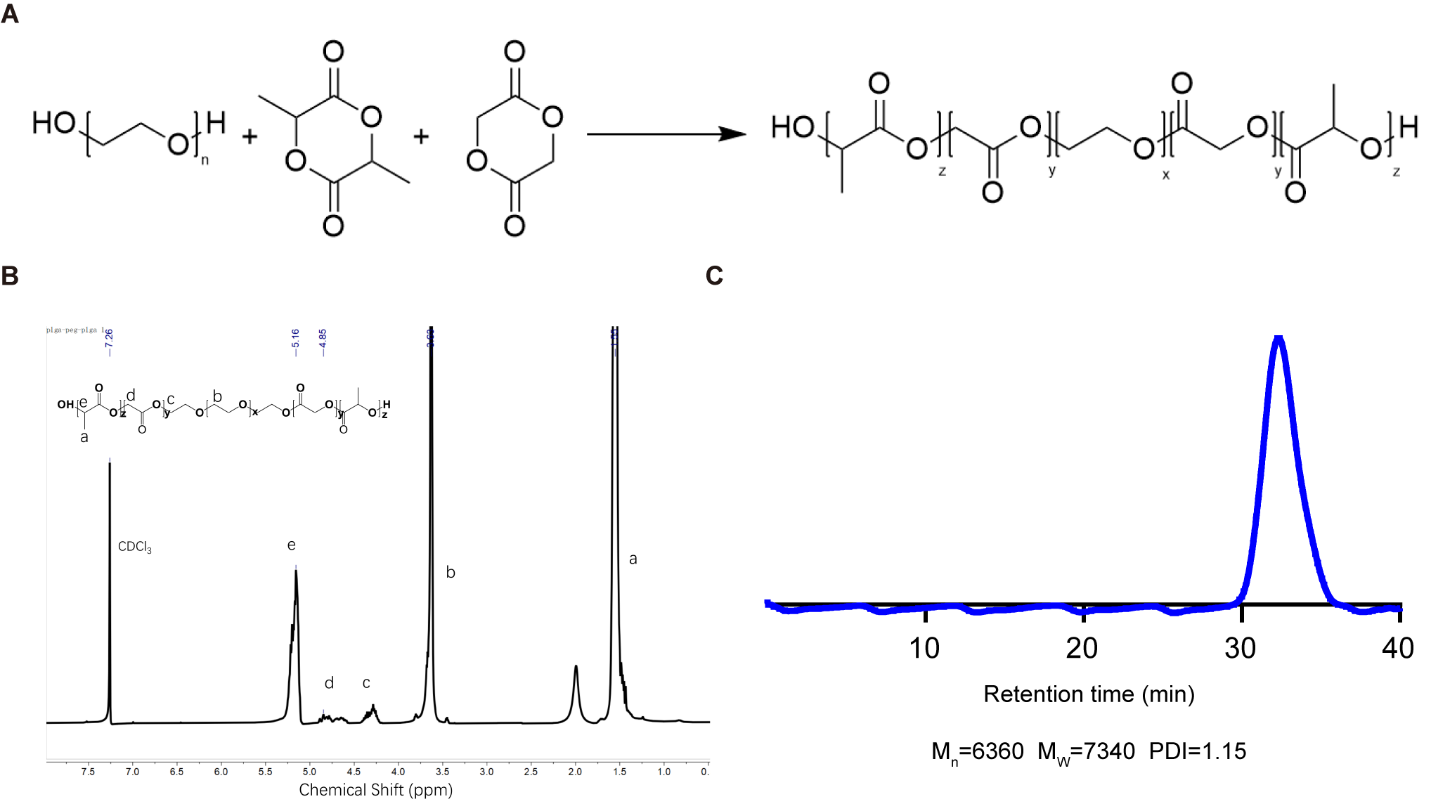


**Figure S6.** A) Synthesis route of PLGA-PEG-PLGA triblock polymer. B) The PLGA-PEG-PLGA structure was determined using Hydrogen nuclear magnetic resonance (H-NMR). C) GPC trace of PLGA-PEG-PLGA.


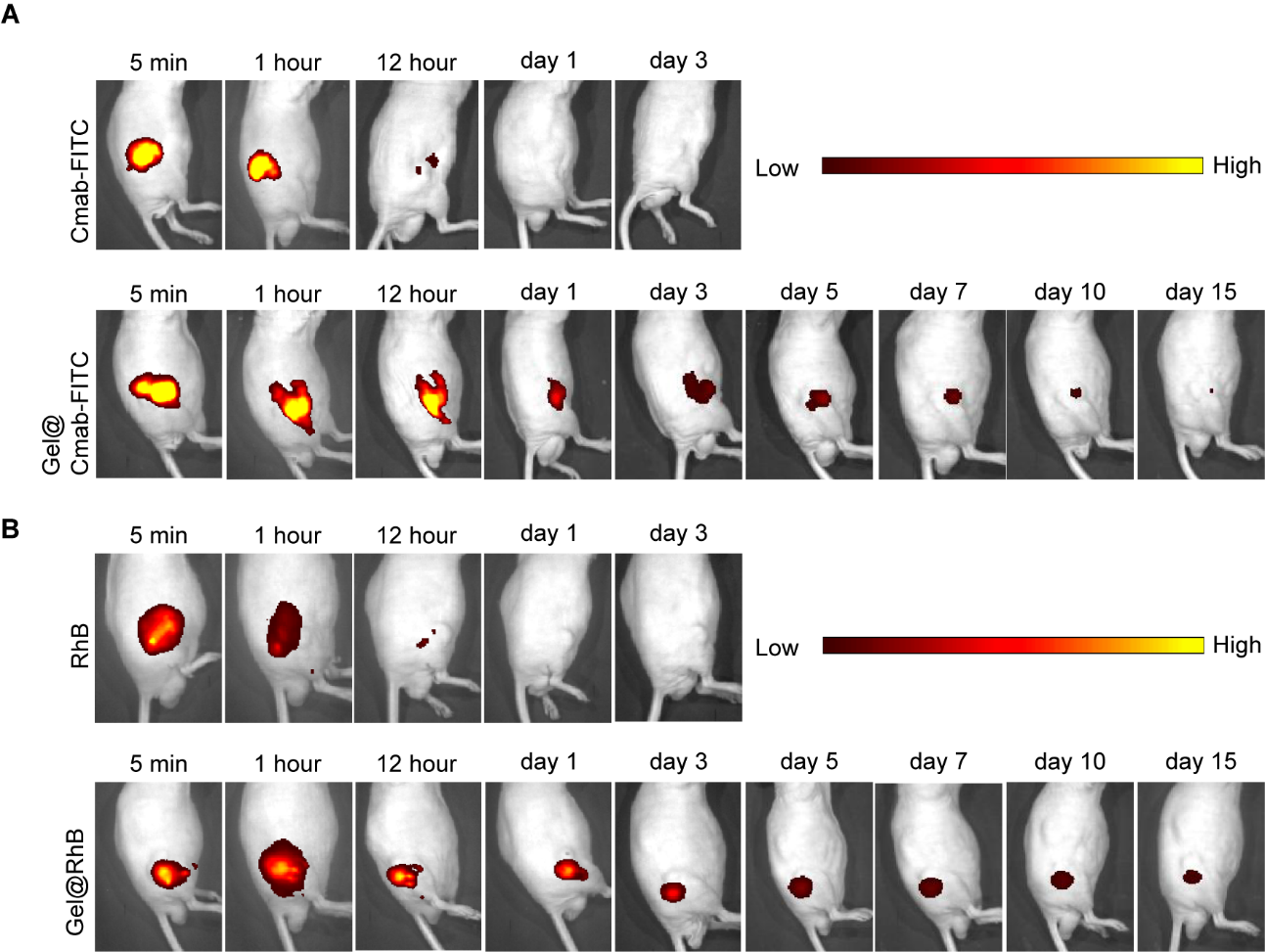


**Figure S7.** A) *In vivo* ﬂuorescence images of free Cmab-FITC and Gel@Cmab-FITC captured by IVIS in diﬀerent release media. B) *In vivo* ﬂuorescence images of free RhB and Gel@RhB captured by IVIS in diﬀerent release media (n = 3).


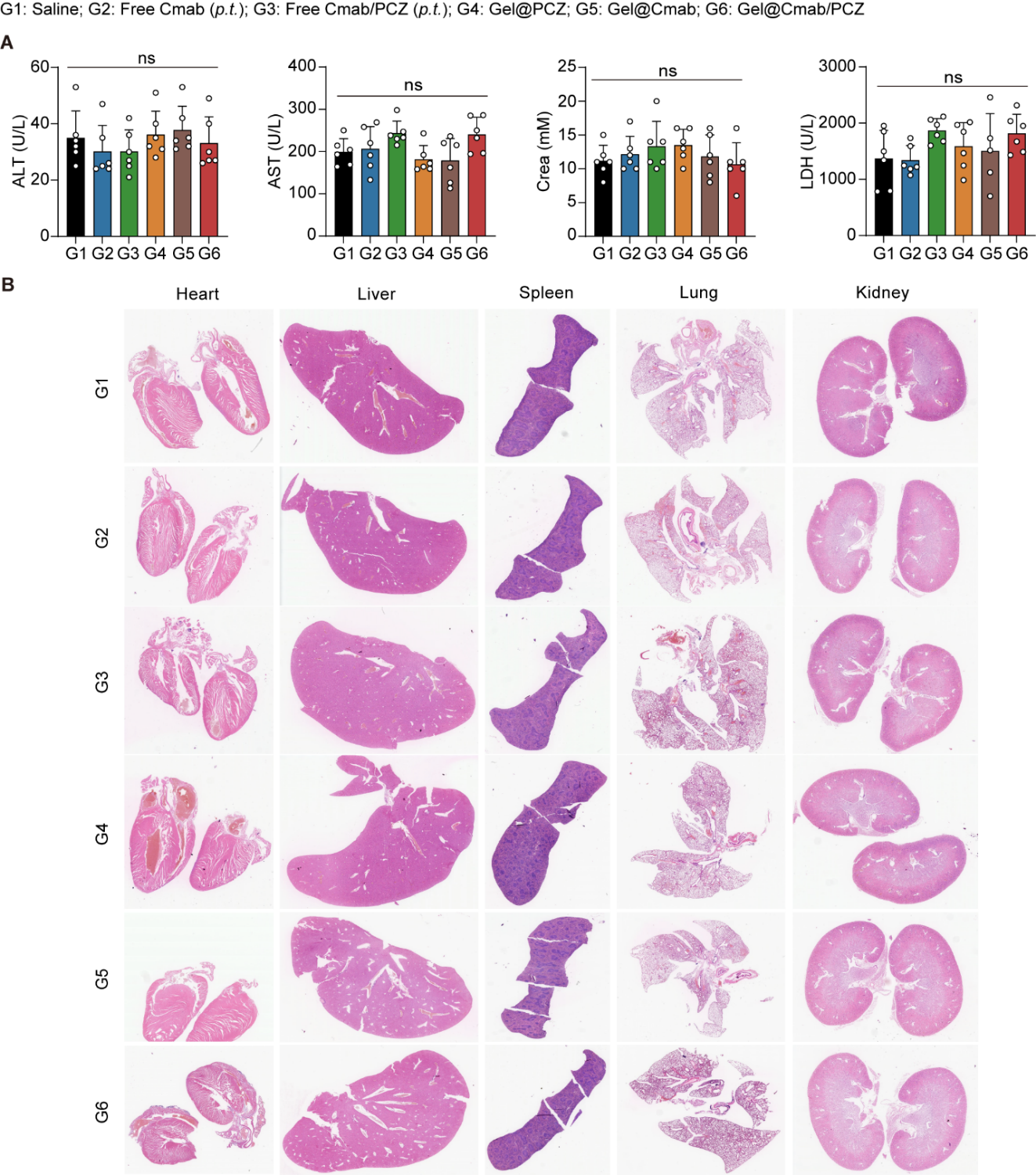


**Figure S8.** *In vivo* biological safety assessment of Gel@Cmab/PCZ. A) Serum ALT, AST, CRE, and LDH tested on nude mice received different treatments. B) Representative H&E-stained images of heart, liver, spleen, lung, and kidney from mice in indicated groups (n = 6). Data are presented as mean ± S.D. Statistical significance was calculated *via* the one-way ANOVA (A).


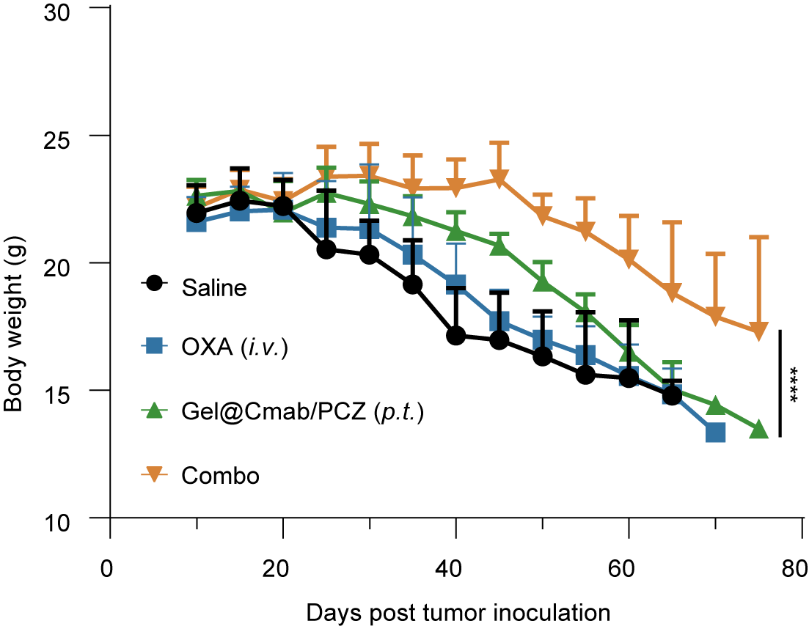


**Figure S9.** Body weight (g) of orthotopic HT29 tumor model from various groups of mice (n=5). Data are presented as mean ± S.D. Statistical significance was calculated *via* the two-way ANOVA. ****P < 0.0001.


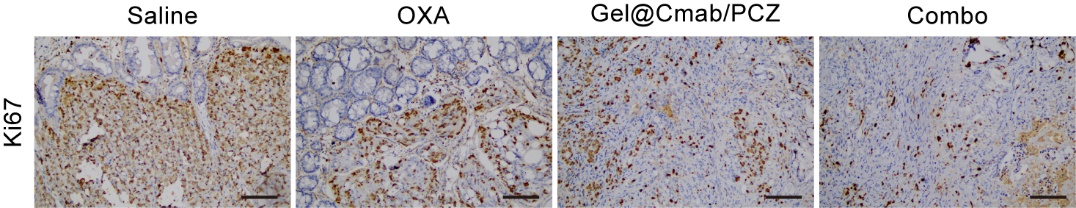


**Figure S10.** The representative of Ki67 staining images of orthotopic HT29 colon cancer tumor tissues after different treatments. Scale bars, 50 μm.


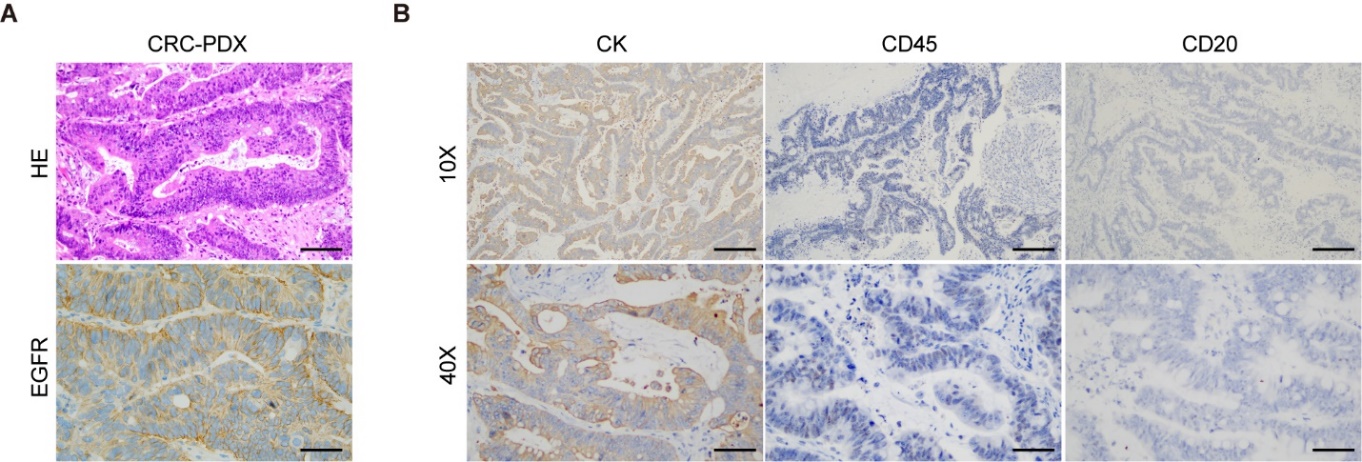


**Figure S11.** A) Representative images of H&E staining and immunohistochemical (IHC) detection of EGFR expression in patient-derived xenograft (PDX) tumor samples. Scale bar, 50 μm. B) Carcinomas were confirmed by IHC pancytokeratin^+^/CD45^-^/CD20^-^. B cell lymphomas excluded from the studies were IHC pancytokeratin^-^/CD45^+^/CD20^+^. Scale bar, 200 μm (Top), 50 μm (Down).


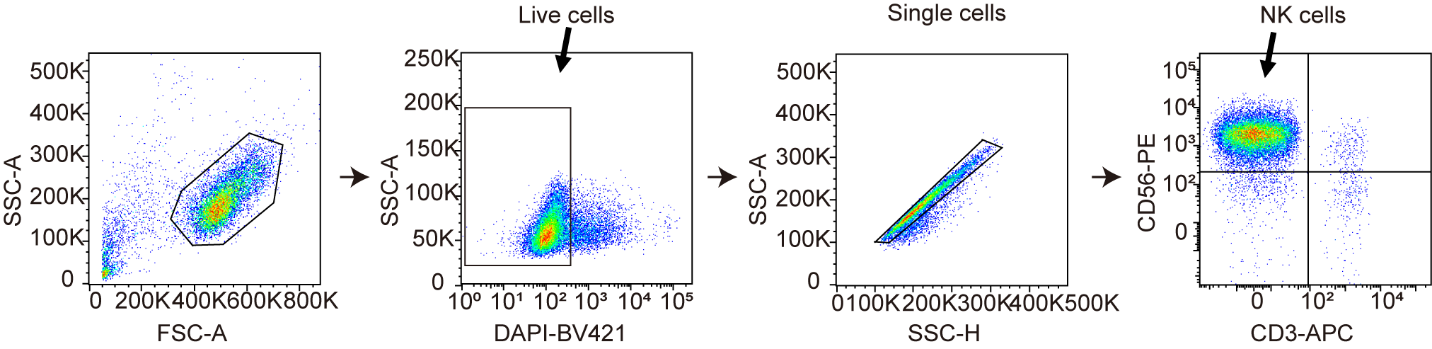


**Figure S12.** Gating strategy used for flow cytometry (FACS) to identify hNK cells in CRC-PDX tissue after treatment.


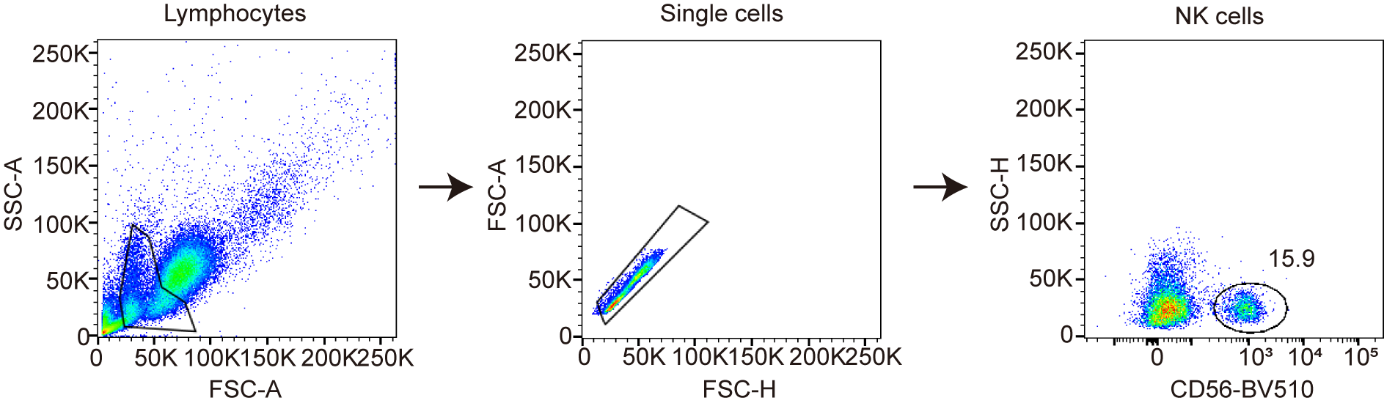


**Figure S13.** Gating strategy used to identify PB-hNK cells by FACs.


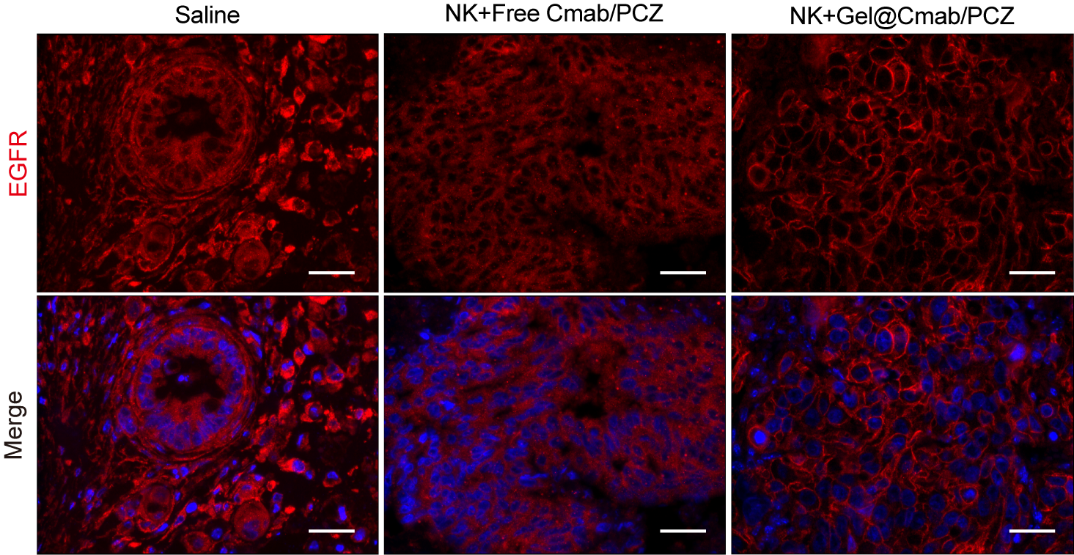


**Figure S14.** Immunofluorescence detection of EGFR localization in CRC-PDX tissue following treatment. Scale bar, 20 μm.


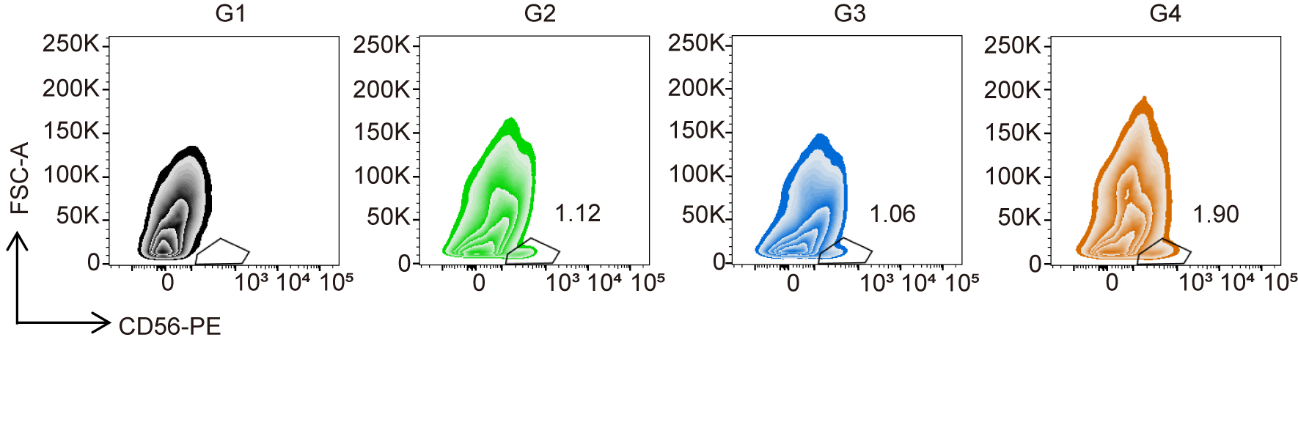


**Figure S15.** Flow cytometry analysis of CD56^+^ NK cell infiltration ratio in CRC-PDX tumor tissues.

**Table S1.** Baseline characteristics of CRC patients received cetuximab treatment.

| **Variable** | **EGFR internalization** | **EGFR membrane localization** |
| --- | --- | --- |
| No. patients | 24 | 13 |
| Age (year) (median, range) | 63.5 (25-74) | 63.0 (47-73) |
| BMI (kg/m^2^) (median, IQR)  ) (median, IQR) | 21.0 (20.2-23.0) | 24.0 (21.5-25.3) |
| Gender |  |  |
| Male (n, %) | 17 (70.8) | 10 (76.9) |
| Female (n, %) | 7 (29.2) | 3 (23.1) |
| Family history (n, %) |  |  |
| Yes | 4 (16.7) | 2 (15.4) |
| No | 20 (83.3) | 11 (84.6) |
| Histology (n, %) |  |  |
| Adenocarcinoma | 22 (91.7) | 13 (100.0) |
| Mucinous | 2 (8.3) | 0 (0.0) |
| T stage (n, %) |  |  |
| T2 | 2 (8.4) | 0 (0.0) |
| T3 | 5 (20.8) | 5 (38.5) |
| T4 | 17 (70.8) | 8 (61.5) |
| N stage (n, %) |  |  |
| N0 | 7 (29.2) | 2 (15.3) |
| N1 | 11 (45.8) | 5 (38.5) |
| N2 | 6 (25.0) | 6 (46.2) |
| M1 (n, %) | 15 (62.5) | 8 (61.5) |
| Clinical Stage (n, %) |  |  |
| Ⅰ | 1 (4.2) | 0 (0.0) |
| Ⅱ | 2 (8.3) | 1 (7.7) |
| Ⅲ | 6 (25.0) | 4 (30.8) |
| Ⅳ | 15 (62.5) | 8 (61.5) |
| Tumor size (cm) (median, IQR) | 4.0 (3.7-5.2) | 3.5 (2.8-5.5) |

*Abbreviations: CRC= colorectal cancer; EGFR= epidermal growth factor receptor; BMI = body mass index; IQR = interquartile range.*

**Table S2.** The number of mice with liver metastasis after different treatments.

| **Group** | **liver metastasis rate (n, %)** |
| --- | --- |
| G1 | 4/5 (80.0) |
| G2 | 3/5 (60.0) |
| G3 | 3/5 (60.0) |
| G4 | 0/5 (0.0) |

*Abbreviations: G1= Saline; G2= NK+Free Cmab/PCZ; G3= NK+Free Cmab; G4= NK+Gel@Cmab/PCZ.*
